# Breeding Bird Community Continues to Colonize Riparian Buffers Ten Years after Harvest

**DOI:** 10.1371/journal.pone.0143241

**Published:** 2015-12-04

**Authors:** Scott F. Pearson, Jack Giovanini, Jay E. Jones, Andrew J. Kroll

**Affiliations:** 1 Wildlife Research Division, Washington Department of Fish and Wildlife, Olympia, Washington, United States of America; 2 Weyerhaeuser, Federal Way, Washington, United States of America; Centre for Cellular and Molecular Biology, INDIA

## Abstract

Riparian ecosystems integrate aquatic and terrestrial communities and often contain unique assemblages of flora and fauna. Retention of forested buffers along riparian habitats is a commonly employed practice to reduce potential negative effects of land use on aquatic systems. However, very few studies have examined long-term population and community responses to buffers, leading to considerable uncertainty about effectiveness of this practice for achieving conservation and management outcomes. We examined short- (1–2 years) and long-term (~10 years) avian community responses (occupancy and abundance) to riparian buffer prescriptions to clearcut logging silvicultural practices in the Pacific Northwest USA. We used a Before-After-Control-Impact experimental approach and temporally replicated point counts analyzed within a Bayesian framework. Our experimental design consisted of forested control sites with no harvest, sites with relatively narrow (~13m) forested buffers on each side of the stream, and sites with wider (~30m) and more variable width unharvested buffer. Buffer treatments exhibited a 31–44% increase in mean species richness in the post-harvest years, a pattern most evident 10 years post-harvest. Post-harvest, species turnover was much higher on both treatments (63–74%) relative to the controls (29%). We did not find evidence of local extinction for any species but found strong evidence (no overlap in 95% credible intervals) for an increase in site occupancy on both Narrow (short-term: 7%; long-term 29%) and Wide buffers (short-term: 21%; long-term 93%) relative to controls after harvest. We did not find a treatment effect on total avian abundance. When assessing relationships between buffer width and site level abundance of four riparian specialists, we did not find strong evidence of reduced abundance in Narrow or Wide buffers. Silviculture regulations in this region dictate average buffer widths on small and large permanent streams that range from ~22–25 m. Guidelines for this region are within the range of buffers included in our study, in which we observed no evidence for avian species loss or for a decline in species abundance (including riparian associated species).

## Introduction

Riparian ecosystems integrate aquatic and terrestrial communities and often support unique assemblages of flora and fauna [1]. Riparian areas can be more structurally diverse and more productive than adjacent uplands [2–4], and may harbor a greater number of plant and vertebrate species [5–7]. Due to the ecological importance of riparian ecosystems, buffers of standing trees or intact native vegetation are often left between harvested stands and aquatic environments to reduce potential negative effects of timber harvest or other forms of land use [8–10]. Buffers may support natural processes and functions of the aquatic system (e.g., shading, sedimentation interception, inputs of large wood and leaf litter) [11]; retain aquatic species and communities [12–14]; protect riparian flora and fauna [15, 16], and support exchange of nutrients from aquatic to terrestrial systems [17–19]. Also, buffers may serve as dispersal corridors and counteract problems associated with landscape fragmentation [20, 21] but see [22, 23].

Riparian buffer width is the primary variable influenced by state and provincial guidelines in the United States and Canada when buffering riparian systems from the effects of silviculture practices in forested landscapes [24, 25]. Despite numerous research efforts to evaluate buffer effectiveness in conserving species and ecological processes, considerable variation in buffer width guidelines exists among jurisdictions [25, 26]. In part, this uncertainty is due to substantial variation in biotic and abiotic responses [27]. For example, in a meta-analysis using data from 397 comparisons of species abundance in riparian buffers and unharvested riparian sites, responses of terrestrial species were not consistent between taxonomic groups [28]. In general, bird and arthropod abundances increased in buffers relative to unharvested areas, whereas amphibian abundance decreased [28].

To examine effects of riparian buffer width on avian community richness and abundance in forested landscapes, investigators evaluated changes in species richness with distance from the stream in unharvested forests [29] and correlated buffer width with species abundance and richness after timber harvest [30, 31]. Other studies used an experimental approach to examine effects of buffer width on species and communities [32, 33] and effects of tree harvest within riparian habitats [34]. To date, few studies of species responses to buffer width have documented long-term effectiveness of the buffer in maintaining presence and/or abundance of riparian associated species; quantitatively identified riparian associates and the effectiveness of the buffer in maintaining those species; or addressed variation in detection that was confounded with treatment [35, 36] and consequently resulted in apparent effects [37].

In the precursor to this study, Pearson and Manuwal [38] described immediate post-harvest (1–2 year post-harvest) responses to two riparian buffer treatments: a uniform width buffer and a wider, and more variable, width buffer. Both buffer prescriptions were created during clearcut logging of uplands adjacent to small streams in western Washington, USA (S1 Fig). Here, we revisited study sites (~10 years post-harvest) and used the same Before-After-Control-Impact (BACI) experimental approach to examine longer-term effects on the avian community. Specifically, we evaluated buffer treatment effects on species abundance and richness, local extinction (site-level species loss) and turnover, and similarity in community composition between treatments and controls. At the species level, we examine treatment effects on occupancy and abundance with a focus on species associated with riparian habitats. In a second analysis that was not conducted with the short-term data, we took advantage of the variability in buffer width both within and among treatments to examine the relative influence of riparian buffer width and vegetation (trees and shrubs) on species occupancy and abundance. Doing so allowed us to identify thresholds in the effects of buffer width on species associated with riparian habitats.

## Methods

### Study Area & Experimental Design

The experiment was conducted on the west side of the southern Cascade Mountains and in the coast range of the state of Washington, USA (S1 Fig). All sites were located in the Western Hemlock forest zone [39]. Douglas-fir (*Pseudotsuga menziesii*), western hemlock (*Tsuga heterophylla*) and western red cedar (*Thuja plicata*) are dominant conifer species in this region. Deciduous tree species are not common in this zone except in recently disturbed sites, talus slopes, and riparian habitats. Riparian habitats are often dominated by red alder (*Alnus rubra*) and big-leaf maple (*Acer macrophyllum*) in early seral stages and by western hemlock and red cedar in later stages. The region is characterized by ridges and steep valleys and the climate consists of warm dry summers and cool wet winters. Lands used in this research were owned by the State of Washington, the City of Seattle, and private timber companies (see Acknowledgments) and managed primarily for production of even-aged Douglas-fir forests. The majority of the landscape, including study stands, has been harvested once or twice previously.

We used a Before-After-Control-Impact experimental design [40] to examine bird response to narrow and wider, forested riparian buffers left along streams after clearcut harvest of the uplands. In 1991 and 1992, we selected 18 sites along small streams and assigned sites randomly to treatments (S1 Fig). Site selection used the following criteria: low elevation (< 620 m); second growth forest (45–65 yrs old); dominated by Douglas-fir and western hemlock in the uplands; second and third order streams [41]; predominantly coniferous riparian canopy with deciduous tree component; at least 500 m in stream length and 300 m wide (150 m wide on each side of the stream) to accommodate point counts (see Avian Sampling below); and a common management history (e.g., thinned at the same time in the past) and likely to be harvested as a single unit in the future. Sites ranged in size from ~33–50 ha, and each site was located along a different stream. Study sites were owned and managed by Champion Pacific Timberlands, City of Seattle, International Paper, Hampton Tree Farms, Plum Creek Timber, The Campbell Group, Washington State Department of Natural Resources and The Weyerhaeuser Company. All landowners provided permission for sampling to occur after reviewing all sampling procedures and experimental manipulations. In addition, all landowners participated in the study by either implementing the treatments on their lands as prescribed, as part of their timber management operations, or by reserving control sites from harvest.

The experimental design consisted of three treatments each with six replicates: 1) forested control sites with no harvest; 2) sites harvested according to 1992 Washington State Forest Practices regulations that consisted of clearcut uplands on each side of the stream with narrow unharvested forest reserves or buffers (Average = 13.1, range = 6.7–25.5m) along each side of the stream (Narrow treatment); and 3) sites harvested with a variable width unharvested buffer reserve that was wider and more variable than the Narrow treatment (Average = 29.9, range = 21.7–40.7 m; Wide treatment). Wide buffered sites were modified to accommodate local features such as seeps and structural components such as snags and down wood. Operators harvested sites in 1994. We collected pre-harvest data in the spring of 1993 from all 18 sites; immediate post-harvest data in the spring of 1995 and 1996; and long-term data approximately 10 years after harvest in 2003 and 2004. Three sites (one in each treatment type) in total were lost to harvest or not available for sampling in 2003–2004, resulting in a reduction in sample size between sampling periods from 18 to 15 sites.

### Avian Sampling

We surveyed the avian community using 15-m fixed radius point counts [42]. All birds were detected by sight or sound. As a result, no birds were captured or handled for this study and this study did not include state or federally listed species. In each site, we established 10 riparian point count stations along the edge of the stream with five stations spaced evenly on each side of the stream. The center of each riparian station was located 15 m (perpendicular distance) from the usual high water line, 100 m from other stations and at least 50 m from the edge of the study site. Ten additional point count stations were located parallel and 100 m upslope from the riparian stations in the adjacent uplands. Data from the upland stations were only used in the pre-harvest year to identify birds that were more abundant in the riparian habitat [38]. Reference flags were placed 15 m to each side of each station. Small radius point counts allowed us to examine differences in bird abundance along narrow strips of potential habitat post-harvest and also to reduce detection issues associated with adjacent stream noise. Point counts rather than strip transects were used because it would have been difficult to both walk and observe birds in the dense vegetation and rugged terrain. Censuses usually started within 30 minutes of dawn and were completed within 5 hours. Upon arriving at a survey point, observers remained stationary and quiet for a minimum of 1 minute to allow birds to settle and then recorded all birds heard or seen during a 6-minute period. To avoid biases among observers, observers were rotated among the 18 study sites. To avoid biases associated with visiting riparian or upland sites first, we alternated travel routes. Each site was visited 6 times between mid-April and late-June. The surveys were evenly spaced throughout the breeding season to account for differences in breeding phenology among species. We did not conduct surveys during heavy precipitation or high winds. Every attempt was made to avoid counting individual birds more than once.

### Sampling Habitat Before & After Harvest

We measured habitat covariates in 15-m^2^ square plots at each bird point count station (*n* = 10 per site) including counts of Douglas-fir, western hemlock/red cedar, and deciduous tree stems > 10 cm at 1.5 m above the ground (hereafter referred to as DBH or Diameter at Breast Height) and percent cover of shrubs (> 1m tall). To measure buffer width, we used a tape to measure the distance between the mean high water mark and the upland edge of the standing trees at each point count station on all treatment sites in the year following harvest and 10 years after harvest. Upland habitats on both buffer treatments were clearcut leaving approximately two standing trees per 0.40 hectare as required by state law. In most cases, these standing trees were located on the outer edge of the riparian buffer by the land managers and consequently were part of the buffer.

### Data Analyses

For all analyses, detections of Hermit (*Setophaga occidentalis*) and Townsend's (*Setophaga townsendi*) warblers were grouped as one species (hereafter Hermit/Townsend's warbler) because these species hybridize extensively in this region [43] and cannot be distinguished by song in regions of hybridization [44]. In addition, we excluded from all analyses individuals that flew over the site, migrants that did not breed in the area [e.g., Ruby-crowned Kinglet (*Regulus calendula*) and Golden-crowned Sparrow (*Zonotrichia leucophrys*)], and all species not adequately sampled by point counts (grouse, raptors, and waterfowl). We excluded all species with less than 10 detections from analyses, either because these species did not breed on the study sites or because species had large territories that cannot be sampled using small radius point counts (e.g., pileated woodpecker *Dryocopus pileatus*).

For all analyses, we aggregated over all point count stations within a site to obtain one response per site per visit. We made this decision to avoid spatial autocorrelation of point count stations within sites, to help with model convergence by reducing the number of species that are not observed at the analysis level, and because the experimental unit was the site (individual point count stations are sub-samples). All sites sampled the same amount of area. However, given that buffer widths varied between treatments, samples represent bird populations within 30 meters of the stream edge, not birds within the riparian buffer. As a result, all inference about avian responses is made with reference to distance from the stream edge. We used repeated visits to a site within a season to estimate detection probabilities as described below.

We used multispecies site occupancy and abundance models [45–47] to estimate species level covariate effects as well as population level summaries of occupancy and abundance, such as species richness, species similarity, and total abundance. We estimated occupancy dynamics, including species turnover and extinction [48, 49]. For both occupancy and abundance, we fit three models (Table 1). In the *Design model*, we modeled the treatment effect as a categorical covariate. In the *Covariates* and *Random Effects* models, we use variation in buffer width both within and among treatments to examine effects of buffer width on abundance and occupancy while ignoring treatment assignments (see Table 2 for the distribution of all site buffer widths). We plotted these estimates against buffer width to determine if a threshold existed in the association. Following others [48, 50], we do not account for the contribution of unobserved species in our population estimates, instead conditioning on the set of observed breeding species in our study.

**Table 1:** Models used to assess avian occupancy and abundance responses to experimental riparian buffer width prescriptions, 1993–2004, Washington, USA.

Model name	Model portion	Notation
*Design*	Process (occupancy/abundance)	$\begin{array}{l} logit\left(\psi_{i,j,k}\right)\;\text{or}\;log\left(\lambda_{i,j,k}\right)=\alpha_{0i}+\alpha_{0k}+\alpha_{1i}\cdot Year.1995_{j}+\alpha_{2i}\cdot Year.1996_{j}+\alpha_{3i}\cdot Year.2003_{j}+\\ \alpha_{4i}\cdot Year.2004_{j}+\alpha_{5i}\cdot Narrow_{k}+\alpha_{6i}\cdot Wide_{k}+\alpha_{7i}\cdot Year.1995_{j}\cdot Narrow_{k}+\\ \alpha_{8i}\cdot Year.1996_{j}\cdot Narrow_{k}+\alpha_{9i}\cdot Year.2003_{j}\cdot Narrow_{k}+\alpha_{10i}\cdot Year.2004_{j}\cdot Narrow_{k}+\\ \alpha_{11i}\cdot Year.1995_{j}\cdot Wide_{k}+\alpha_{12i}\cdot Year.1996_{j}\cdot Wide_{k}+\\ \alpha_{13i}\cdot Year.2003_{j}\cdot Wide_{k}+\alpha_{14i}\cdot Year.2004_{j}\cdot Wide_{k} \end{array}$
	Observation	$\begin{array}{l} logit\left(p_{i,k,j,l}\right)=\beta_{oi}+\beta_{1i}\cdot Year.1995_{j}+\beta_{2i}\cdot Year.1996_{j}+\beta_{3i}\cdot Year.2003_{j}+\\ \beta_{4i}\cdot Year.2004_{j}+\beta_{5i}\cdot Trt.Narrow.det_{k,j}+\beta_{6i}\cdot Trt.Wide.det_{k,j}+\beta_{7i}\cdot Date_{j,k,l}+\\ \beta_{8i}\cdot Date_{j,k,l}^{2} \end{array}$
*Covariates*	Process (occupancy/abundance)	$\begin{array}{c} logit\left(\psi_{i,k,j}\right)\;\text{or}\;log\left(\lambda_{i,k,j}\right)=\alpha_{oi}+\alpha_{ok}+\alpha_{1i}\cdot Year.1996_{j}+\alpha_{2i}\cdot Year.2003_{j}+\alpha_{3i}\cdot Year.2004_{j}+\\ \alpha_{4i}\cdot BufferWidth_{k,j}+\alpha_{5i}\cdot Shrub_{k,j}+\alpha_{6i}\cdot DougFir_{k,j}+\alpha_{7i}\cdot Decid_{k,j}+\alpha_{8i}\cdot HemCedar_{k,j}. \end{array}$
	Observation	$\begin{array}{l} logit\left(p_{i,k,j,l}\right)=\beta_{oi}+\beta_{1i}\cdot Year.1996_{j}+\beta_{2i}\cdot Year.2003_{j}+\beta_{3i}\cdot Year.2004_{j}+\\ \beta_{4i}\cdot BufferWidth_{k,j}+\beta_{5i}\cdot Shrub_{k,j}+\beta_{6i}\cdot DougFir_{k,j}+\beta_{7i}\cdot Decid_{k,j}+\beta_{8i}\cdot HemCedar_{k,j}+\\ \beta_{9i}\cdot Date_{j,k,l}+\beta_{10i}\cdot Date_{j,k,l}^{2}. \end{array}$
*Random Effects*	Process (occupancy/abundance)	$logit\left(\psi_{i,k,j}\right)\;\text{or}\;log\left(\lambda_{i,k,j}\right)=\alpha_{oi}+\alpha_{ok}+\alpha_{1i}\cdot Year.2004_{j}.$
	Observation	$\begin{array}{l} logit\left(p_{l,k,j,i}\right)=\beta_{oi}+\beta_{1i}\cdot Year.2004_{j}+\\ \beta_{2i}\cdot BufferWidth_{k,j}+\beta_{3i}\cdot Shrub_{k,j}+\beta_{4i}\cdot DougFir_{k,j}+\beta_{5i}\cdot Decid_{k,j}+\beta_{6i}\cdot HemCedar_{k,j}+\\ \beta_{7i}\cdot Date_{j,k,l}+\beta_{8i}\cdot Date_{j,k,l}^{2}. \end{array}$

**Table 2:** Summary of post-treatment riparian buffer widths by treatment type (*n* = 5 for each treatment type), western Washington, USA, 1996 and 2003.

Site Name	Treatment	Year	Average (m)	Standard deviation
Blue Tick	Wide	1996	32.0	13.9
Blue Tick	Wide	2003	36.1	22.1
Eleven 31	Wide	1996	21.9	10.8
Eleven 31	Wide	2003	21.9	10.4
Ms Black	Wide	1996	31.0	10.7
Ms Black	Wide	2003	28.1	9.3
Ryderwood 860	Wide	1996	21.7	5.1
Ryderwood 860	Wide	2003	21.7	5.1
Side Rod	Wide	1996	34.4	14.1
Side Rod	Wide	2003	40.7	24.9
All wide buffers			29.9	15.5
Eleven 32	Narrow	1996	8.8	4.0
Eleven 32	Narrow	2003	6.7	5.2
Kapowsin	Narrow	1996	14.5	4.0
Kapowsin	Narrow	2003	6.7	4.7
Night Dancer	Narrow	1996	10.4	3.8
Night Dancer	Narrow	2003	9.3	5.4
Potpourri	Narrow	1996	25.5	12.1
Potpourri	Narrow	2003	21.3	6.7
Simmons Creek	Narrow	1996	15.6	8.8
Simmons Creek	Narrow	2003	8.7	5.4
All narrow buffers			13.1	9.1

For occupancy models, we let *z*_*i*,*j*,*k*_ denote true the occupancy status, in which *z*_*i*,*j*,*k*_ = 1 if species *i* in year *j* occupies site *k* or *z*_*i*,*j*,*k*_ = 0 otherwise. The occupancy state is a Bernoulli random variable,*z*_*i*,*j*,*k*_ ~ *Bern*(*Ψ*_*i*,*j*,*k*_), where *Ψ*_*i*,*j*,*k*_is the probability that species *i* in year *j* occupies site *k* We also have detection follow a Bernoulli distribution, *y*_*i*,*j*,*k*,*l*_ ~ *Bern*(*p*_*i*,*j*,*k*,*l*_⋅*z*_*i*,*j*,*k*_), where *y*_*i*,*j*,*k*,*l*_ is 1 if the species *i* in year *j* is detected at site *k* during visit *l* or 0 otherwise and where *p*_*i*,*j*,*k*,*l*_ is the detection probability. Note that under this parameterization, the probability of detecting species *i* during year *j* at site *k* will be zero if it does not occupy site *k*, since *z*_*i*,*j*,*k*_ = 0.

In the *Design model*, we considered the model based on the experimental design, in which detection probability varied by treatment type (Control, Narrow, and Wide treatments) and year (Table 1). For the detection model, the treatment status effect is the treatment at time of measurement. Therefore, in 1993, all sites had control for the detection model. In addition, we included linear and quadratic terms for Julian date (January 1 = 1, December 31 = 365) because avian detection rates are known to vary seasonally [51]. We centered and scaled the date covariate. The terms *α*_0*i*_ and *α*_0*k*_ are random effects for species and site, respectively. Even though there was substantial variability of buffer widths within the Narrow and Wide buffer treatments (Table 2), this analysis allows us to examine how the buffer treatments would act within the context of operational variability of harvest prescriptions.

To examine how species occupancy differed among buffer prescriptions, we estimated treatment effect sizes [52, 53]. In our parameterization, the year × Narrow and year × Wide coefficients compare occupancy of the respective treatments to the Control, and are estimates of the treatment effects on occupancy. After back transformation, these terms are interpreted as the multiplicative change in odds of occupancy. We estimated species richness (*s*), where *nspp* is the total number of species across all sites by year, for treatment and control plots separately as:

$${\hat{s}}_{j,k}=\sum_{i=1}^{i=nspp}\sum_{k=1}^{k=sites}\hat{z}\left(i,j,k\right).$$

To examine the effect of buffer treatment on species richness, we estimated the mean species richness for the three treatment × five year combinations. In addition to estimated species richness, we estimated species similarity both between and among treatment and control sites [45] by calculating the proportion of species that occupy both sites. Species similarity in year *j*, for sites *k*_1_ and *k*_2_,is defined as:

$$S_{j,k_{1},k_{2}}=\frac{2\sum_{i}\left(z_{i,j,k_{1}}\times z_{i,j,k_{2}}\right)}{\sum_{i}z_{i,j,k_{1}}+\sum_{i}z_{i,j,k_{2}}}.$$

Within each year, we estimated the similarity for all pairwise combinations of sites. This set of summary statistics allows us to determine the impact of buffer treatment on species similarity.

We estimated species turnover (τ), the probability that a species chosen at random from the community at time *j* is a species not present at time *j–* 1, and local-extinction rates (ε) as:

$$\tau \left(j\right)=\frac{\sum_{i=1}^{i=nspp}\sum_{k=1}^{k=sites}z\left(i,k,j\right)\times \left[1-z\left(i,k,j-1\right)\right]}{\sum_{i=1}^{i=nspp}\sum_{k=1}^{k=sites}z\left(i,k,j-1\right)}$$

$$\varepsilon \left(j\right)=\frac{\sum_{i=1}^{i=nspp}\sum_{k=1}^{k=sites}\left[1-z\left(i,k,j\right)\right]\times z\left(i,k,j-1\right)}{\sum_{i=1}^{i=nspp}\sum_{k=1}^{k=sites}z\left(i,k,j-1\right)}.$$

The *Covariates model* examined effects of buffer width (the treatment) and vegetation covariates on occupancy for harvested sites (Table 1). Observations from the pre-treatment year and all control sites were not included in this analysis. The detection model included effects of year, average buffer width (based on 10 measurements) at each site (BufferWidth), percent shrub cover (Shrub), number of Douglas-fir stems > 10 cm DBH (DougFir), number of deciduous stems > 10 cm DBH (Decid), and number of western hemlock and western red cedar stems > 10 cm DBH (HemCedar). We included linear and quadratic terms for Julian date. We centered and scaled all continuous covariates.

We constructed the *Random Effects model* to provide site-specific estimates of species richness without any covariate effects except year (Table 1). We used only the 2003 and 2004 data because we were interested in finding a buffer width that matched the control in the longer-term time frame. The detection model included effects of year, average buffer width (based on 10 measurements) at each site (BufferWidth), percent shrub cover (Shrub), number of Douglas-fir stems > 10 cm DBH (DougFir), number of deciduous stems > 10 cm DBH (Decid), and number of western hemlock and western red cedar stems > 10 cm DBH (HemCedar). We included linear and quadratic terms for Julian date. We centered and scaled all continuous covariates. In the Random Effects model, we did not include either buffer width or vegetation effects because we did not want to ‘force’ a relationship between buffer width and occupancy.

For abundance models, we fit a multispecies version of the N-mixture model [47, 54]. This model is a natural extension of the single species N-mixture model [55, 56] and the multispecies occupancy model [45]. We let *n*_*i*,*j*,*k*,*l*_ be the number of individuals of species *i* in year *j* that are detected at site *k*, and during visit *l*. We define *N*_*i*,*j*,*k*_ as the unobserved site level abundance, assumed constant over visits. We then modeled the observed count, *n*_*i*,*j*,*k*,*l*_ as a Binomial(*N*_*i*,*j*,*k*_, *p*_*i*,*j*,*k*,*l*_) random variable. Following Royle [55], we assume the site level abundance *N*_*i*,*j*,*k*_ follows a Poisson (*λ*_*i*,*j*,*k*_) distribution (Table 1). Abundance covariates are incorporated in the model by assuming that the log-transform of *λ*_*i*,*j*,*k*_ is described by a linear function of the covariates. Detection probability is modeled similarly, where we assume that the logit transform of *p*_*i*,*j*,*k*,*l*_ is a linear function of the covariates.

As with the occupancy model, the year × Narrow and year × Wide coefficients compare abundance of the respective treatments to the Control, adjusting for differences due to year. After back transformation, a treatment contrast of 1 indicates that abundance was equal across treatments.

We estimated the total abundance of all individuals for all species that occupy a site for treatment and control plots separately as:

$$Total\;{\hat{N}}_{j,t}=\sum_{i=1}^{i=nspp}\sum_{k=1}^{k=sites}{\hat{N}}_{i,j,k},$$

where *nspp* is the total number of species across all sites and t is an indicator variable for treatment type. This estimate represents the total number of individuals across all species, where abundance for each species is adjusted by a species-specific detection probability. Finally, we wanted to determine at what buffer width abundance of riparian-associated species and total avian abundance were similar to abundance in the Control sites. To estimate these quantities for each site, we averaged the posterior medians of total abundance and species richness over the years in the study. The resulting means were plotted vs. buffer width of the site.

To examine the association of buffer width and vegetation covariates with species richness and total abundance in the *Covariates model*, we used average predictive comparisons [57] to quantify directly associations (and uncertainty) between predicted species richness and predicted total abundance with each model covariate. Predictive comparisons evaluate the difference in expected response for a unit difference in an input covariate, using the fitted model, and averaging over the distribution of all other covariates. Following Jones et al. [58] and Kroll et al. [59], we extend this approach to species richness and total abundance by summing over the species-specific predictions to obtain averaged expected differences in species count. For dataset (*x*,*y*)_*j*_, *j* = 1,…,*n*, we denote our input of interest *u*, and all other inputs *v*, such that *x = (u*,*v)*, where n is the number of sites. We let *i* = 1,…,*N*, be the index of species, where N is the total number of observed species. We estimated the average predictive comparison for species richness using the following equation:

$${\hat{\Delta }}_{u}=\frac{\sum_{j=1}^{n}\sum_{k=1}^{n}\sum_{s=1}^{S}w_{jk}\sum_{i=1}^{N}\left(E(y|u_{k},v_{j},\theta^{S})-E(y|u_{j},v_{j},\theta^{S})\right)sign(u_{k}-u_{j})}{\sum_{j=1}^{n}\sum_{k=1}^{n}\sum_{s=1}^{S}w_{jk}(u_{k}-u_{j})sign(u_{k}-u_{j})}$$

Let *θ^s^* be a set of *s* = 1,…,*S* simulations were sampled from the posterior distribution. Let *w*_*jk*_ be a weight that reflects how likely a transition from *u*_*j*_ to *u*_*k*_ when *v = v*_*j*_. We calculated predictive comparisons for all model inputs, treating each in turn as the input of interest. Standard errors for ${\hat{\Delta }}_{u}$ are estimated following Gelman and Pardoe [57], and account for uncertainty in model parameter estimates while treating all covariates as fixed.

For all of the hierarchical community models, we assume that the species-specific effects for a given parameter are drawn from a common normal distribution, e.g., that $\alpha_{1,i}\sim N\left(\mu_{1},\sigma_{1}^{2}\right)$ for parameter α_1_ of species *i*, where the mean and variance of *α*_1,i_ are population-level hyper-parameters. This population-level distribution provides a summary of community response, both in terms of the mean behavior as well as the variability in behavior. The extent to which information is shared across species depends on both the degree of uniformity across the population, as estimated by the population-level parameters, and the amount of information available for each species. For species with little information, those with low detection probabilities, estimates will tend to shrink toward the population mean value. To account for the fact that the same sites are sampled in multiple years, we included a site level random effect, $\alpha_{0k}\sim N\left(0,\sigma_{k}^{2}\right)$. This approach is analogous to a ‘compound symmetric’ correlation structure for years within a site [60].

We fit our model using JAGS [61] called from R version 2.15.2 [62] using the ‘jags’ function in package R2jags version 0.03–08 [63]. For all models, we ran three Markov chains of length 400,000 with a burn-in period of 200,000 and 1/50 thinning. We provide all code for the models in the supplementary material (S1 and S2 Text). We assessed convergence using the Gelman-Rubin statistic [64] and visual inspection of the chains, with both measures indicating a reasonable assumption of convergence. To assess consistency between our models and data, we used posterior predictive checks [65]. We did not find any evidence of lack of fit in the models (S2 Text). We provided details and an example for the posterior predictive checks in the supplementary material.

## Results

**Fig 1:**
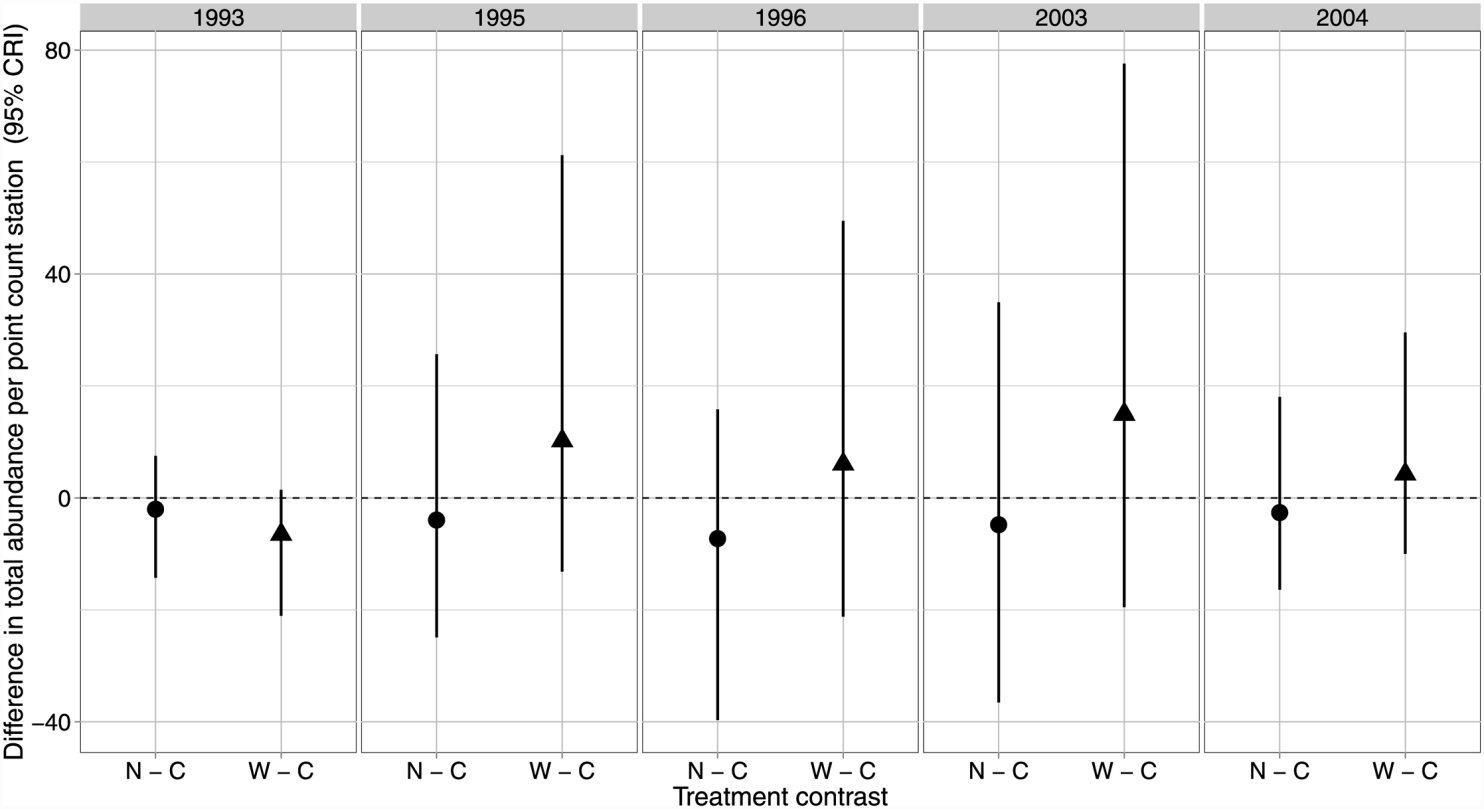
Species abundance contrasts. Contrasts (95% credible interval) for the difference in the total number of birds of all species per point count station between the control (C) and each treatment (N, Narrow, and W, Wide) before harvesting (1993) immediately following (1995, 1996), and 10 years post (2003, 2004) in western Washington, USA. Each treatment had 5 experimental units (*n* = 15).

Using the *Design model* (Table 1), we found broad overlap in credible intervals associated with our estimates of total bird abundance for controls and treatments for the pre- and post-harvest time periods (Fig 1). Within sampling year, we found less variation among treatment point estimates of abundance relative to the uncertainty associated with those estimates (Fig 1). Note that the credible intervals are wide indicating uncertainty about parameter estimates. In general, avian abundance moved up and down between time periods similarly among all sites post-treatments (Fig 1).

**Fig 2:**
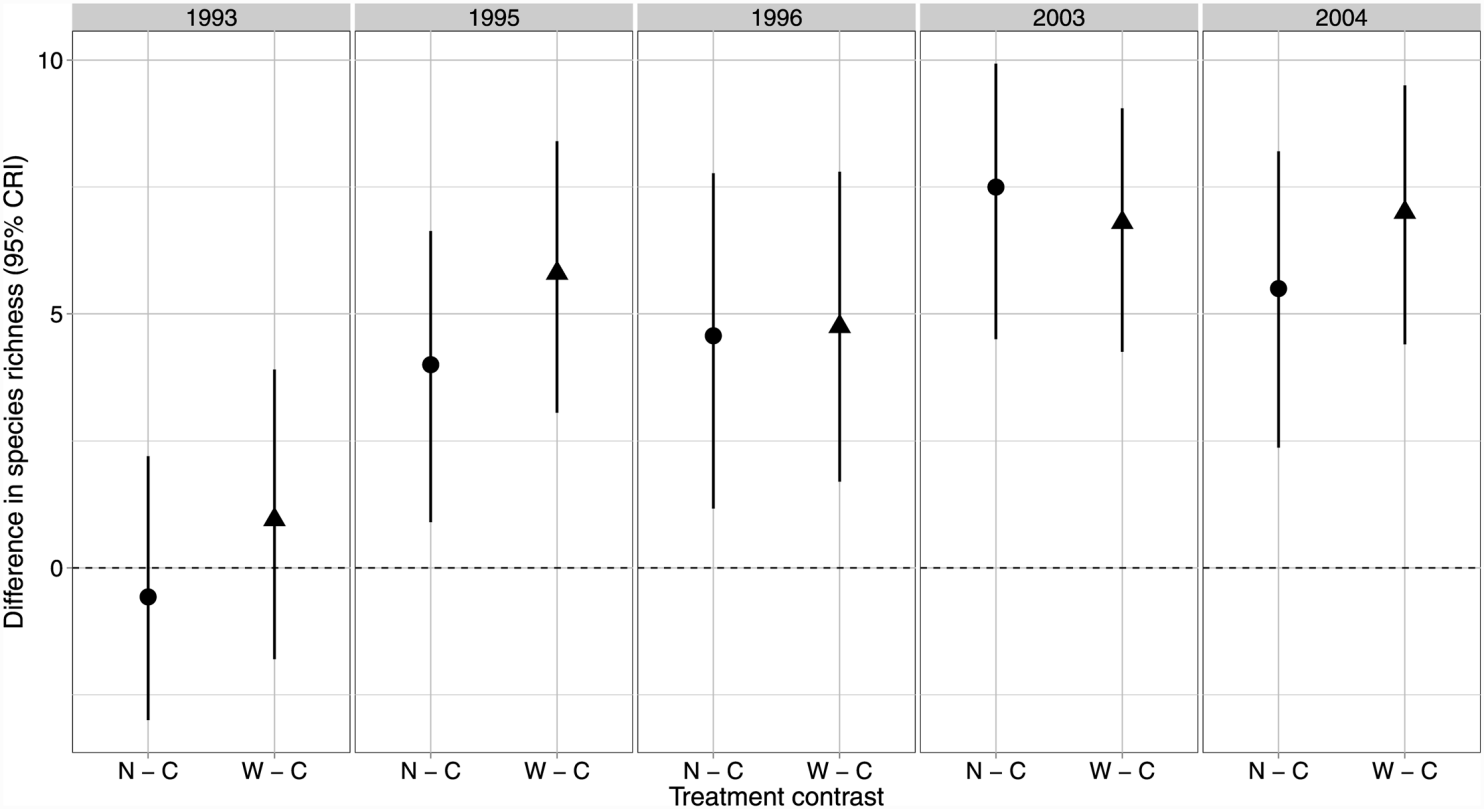
Species richness contrasts. Contrasts (95% credible interval) for the difference in the median number of species per site between the control (C) and each treatment (N, Narrow, and W, Wide) before harvesting (1993) immediately following (1995, 1996), and 10 years post (2003, 2004) in western Washington, USA. Each treatment had 5 experimental units (*n* = 15).

**Fig 3:**
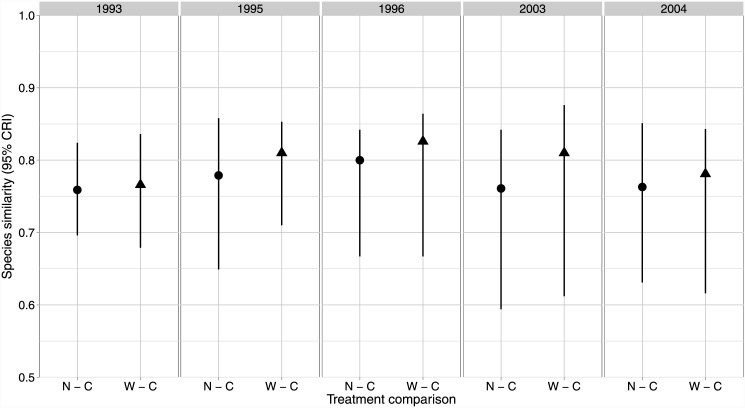
Species similarity contrasts. Contrasts (95% credible interval) for the median species similarity (%) per site between the control (C) and each treatment (N, Narrow, and W, Wide) before harvesting (1993) immediately following (1995, 1996), and 10 years post (2003, 2004) in western Washington, USA. Each treatment had 5 experimental units (*n* = 15). Species similarity is an estimate of the percent of species shared by two treatments in a given year.

**Fig 4:**
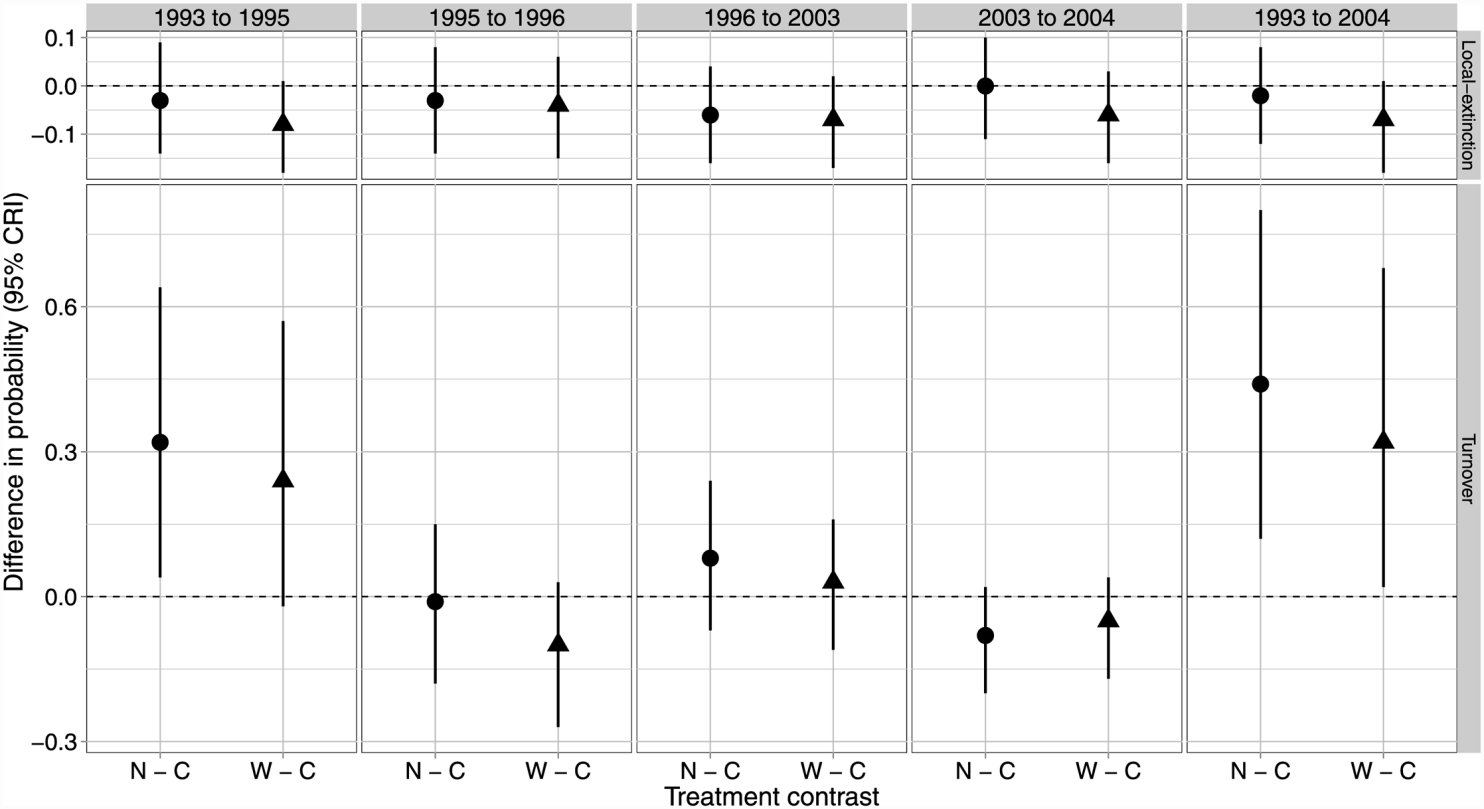
Local extinction and turnover contrasts. Contrasts (95% credible interval) for the difference in local extinction and turnover probabilities between pairs of years by treatment (C, Control; N, Narrow; and W, wide) in western Washington, USA, 1993 (pre-harvest), 1995–1996, and 2003–2004. Each treatment had 5 experimental units (*n* = 15). Turnover is the probability that a species selected at random from a treatment at time *t* is a “new” species. Local-extinction is the probability that a species that occupied a treatment in time t did not occupy the treatment in time *t* + 1.

Across all years and treatments, median estimates of species richness ranged from approximately 13–24 bird species with lower pre-harvest richness on all treatments. Estimates of post-harvest richness change little on Control sites relative to pre-harvest levels (Fig 2), while both treatments exhibit a similar 31–44% increase post-harvest (Fig 2). Richness estimates on both treatments continued to increase by about 1–2 species between the immediate post-harvest survey (slight credible interval overlap between treatments and controls) and the 10 year post-harvest survey (no credible interval overlap between treatments and control; Fig 2). Species similarity among treatments overlapped broadly before and after harvest (Fig 3). Site-level estimates of species local-extinction rates were almost identical between treatments and controls regardless of the time periods compared (Fig 4). Species turnover was also almost identical for the two buffer treatments and controls for all years compared, except when comparing the pre-harvest sample to the 10 year post-harvest sample where little overlap in credible intervals occurred between the Narrow treatment and the control (Fig 4) and with much higher turnover on both treatments (63% and 74%) relative to the controls (29%).

**Fig 5:**
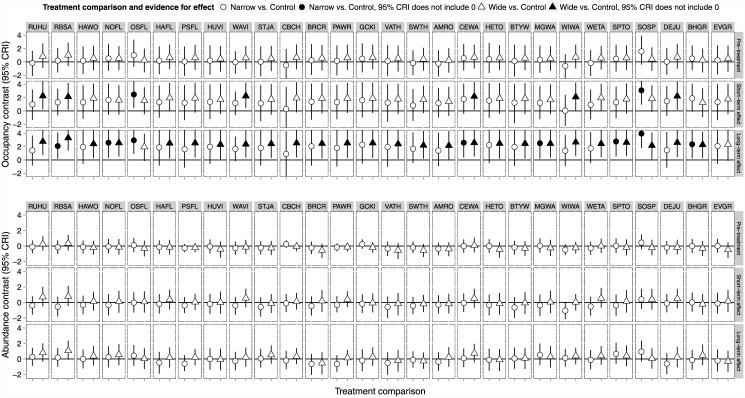
Species level abundance and occupancy contrasts. Contrasts (95% credible interval) for occupancy (top) and abundance (bottom) between the control and each treatment (Wide and Narrow forested riparian buffers) before harvesting, immediately following, and 10 years post-harvest in western Washington, USA, 1993 (pre-harvest), 1995–1996, 2003–2004. A point estimate of 1 suggests that a given species has ~2.7 times greater odds to occupy the treatment as the control or is 2.7 times as abundant on the treatment than the control. A solid symbol indicates 95% CRI do not overlap 0; an open symbol indicates that the 95% CRI does include 0. Species acronyms are provided in Table 2. Note that differences in occupancy (solid symbols) do not become evident until 10 years post-harvest.

Pre-harvest, estimated probability of species-level occupancy was similar for the control and each treatment (95% credible intervals for differences broadly overlapped 0 for all species; Fig 5). Post-harvest, 7 and 21% of the species had increased probabilities of occupancy (95% credible intervals associated with the probability of species occupancy did not overlap zero) in the short-term and 29 and 93% species had increased probabilities of site occupancy in the long-term on the Narrow and Wide buffer treatment, respectively (Fig 5). Probability of site occupancy did not decrease for any species (Fig 5). Probability of occupancy increased for both interior conifer forest species like the golden-crowned kinglet (*Regulus satrapa*) and for species associated with edge and more open habitats like the northern flicker (*Colaptes auratus*). We found no clear evidence for species-level differences (all credible intervals overlapped zero) in abundance between buffer treatments and the Control for either time period assessed (Fig 5).

Across all years (1993, 1995–1996, and 2003–2004) and treatments (Control, Wide and Narrow buffer), we had 28 species detected at least 10 times total for a total of 2064 detections (S1 Table). A few species constituted the majority (60%) of the detections including the Pacific wren (*Troglodytes pacificus*), Pacific-slope flycatcher (*Empidonax difficilis*), chestnut-backed chickadee (*Poecile rufescens*), Wilson’s warbler (*Cardellina pusilla*), Swainson’s thrush (*Catharus ustulatus*), and American robin (*Turdus migratorius*). For reference, we provide the effect (95% credibility interval) of three riparian buffer treatments on detection and capture probabilities for all 28 species (S2 and S3 Tables).

Average riparian buffer was 13.1 (±9.1 SD) and 29.9 m (±15.5 SD) on the Narrow and Wide treatments, respectively, with considerable within-treatment variation (Table 2). The widest buffer on the Narrow treatment (25.5±12.1 SD) overlapped the narrowest buffer on the Wide treatment (21.7±5.1 SD). In general, treatments resulted in greater shrub cover and number of deciduous and Douglas-fir trees in the riparian and fewer western hemlock and western red cedar trees 10 years post-harvest (Table 3) than the control.

**Table 3:** Summaries (average and standard error) of four vegetation covariates, percent shrub cover and total number of stems >10 cm in diameter for all deciduous trees combined, Douglas-fir, and western hemlock and western red cedar combined, by treatment type (*n* = 5 for each treatment type), western Washington, USA, 1993, 1996, and 2004.

Treatment and year	Shrub cover	SE	Deciduous	SE	Douglas-fir	SE	Western hemlock/western red cedar	SE
Control 1993	15.7	2.8	97.8	25.8	13.8	1.0	57.6	22.7
Control 1996	19.8	3.9	74.4	23.1	17.4	3.3	80.8	23.2
Control 2004	4.3	1.9	61.2	36.0	26.4	5.2	121.4	16.1
Narrow 1993	14.0	2.9	78.4	10.6	22.4	6.6	79.8	22.8
Narrow 1996	18.0	4.1	99.6	14.3	31.4	16.9	73.4	18.9
Narrow 2004	6.5	2.0	89.4	15.3	44.8	13.0	85.4	22.6
Wide 1993	7.8	3.4	82.8	33.2	29.6	9.2	101.4	25.4
Wide 1996	6.8	3.3	68.8	31.1	11.8	1.9	97.8	18.3
Wide 2004	9.5	1.4	148.8	10.4	42.6	9.5	86.2	15.2

**Fig 6:**
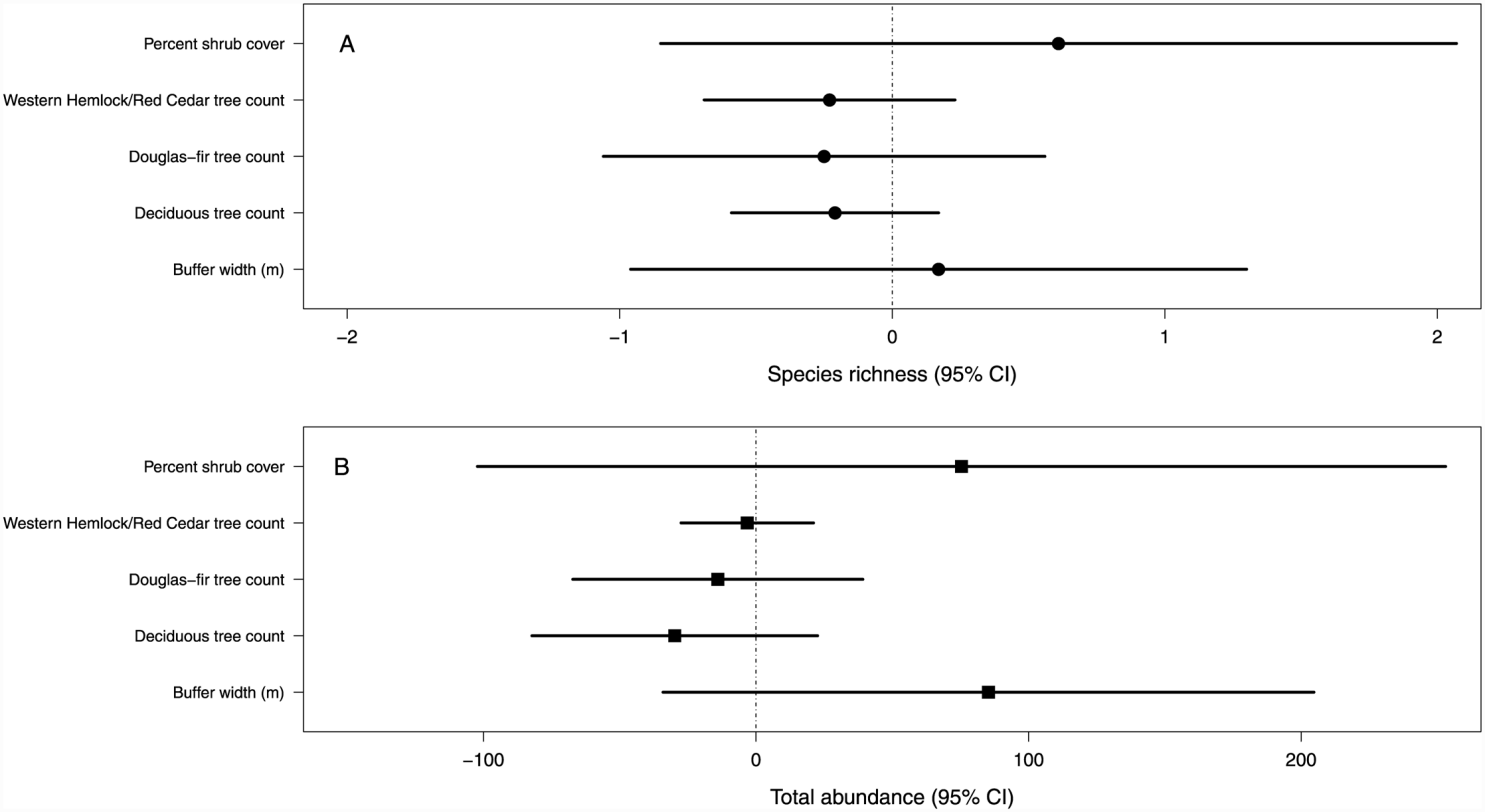
Vegetation and buffer width influences on abundance. Average (95% credible interval) predicted effect (while holding the other 4 covariates at their mean values) of each vegetation (trees and shrubs) and buffer width covariate on species richness (A) and total bird abundance (B).

Using the *Covariates model*, we found no effect of vegetation (deciduous trees, Douglas-fir trees, western hemlock/red cedar trees, and shrubs) or buffer width covariates on species richness or total avian abundance (Fig 6). For buffer width, we found little (16%) overlap between total avian abundance and zero, providing some evidence (84%) for a positive effect of buffer width on avian abundance.

Nearly all credible intervals broadly overlapped zero for relationships between species abundance/occupancy and either buffer width or vegetation covariates (S2 Fig). The few relationships (8 out of 280) where credible intervals did not overlap zero included a positive effect of buffer width on chestnut-backed chickadee abundance; negative effect of deciduous tree density on Pacific-slope flycatcher, chestnut-backed chickadee, golden-crowned kinglet, and dark-eyed junco abundance; positive effect of Douglas-fir tree density on Steller’s jay abundance; negative effect of western hemlock and western red cedar density on Wilson’s warbler abundance; and a positive effect of shrub cover on warbling vireo occupancy (S2 Fig).

**Fig 7:**
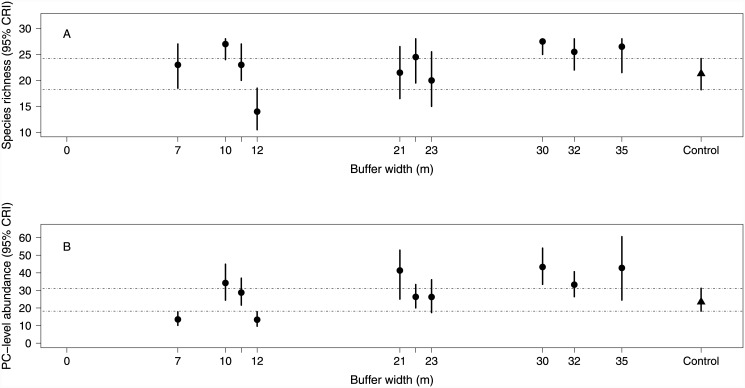
Buffer width influences on species richness and abundance. Estimates (95% confidence interval) of site level species richness (A), and total abundance (B)plotted against site specific buffer width. Estimates were calculated from a model with a random site-level effect but no covariates. Control site species richness and abundance are provided on the right side (triangle) of each graphic. Intervals are confidence intervals, and not credibility intervals. Estimates for all sites were averaged across 1995–2004. Horizontal lines extending from the upper and lower bounds of the confidence intervals for the control sites are provided as reference lines.

**Fig 8:**
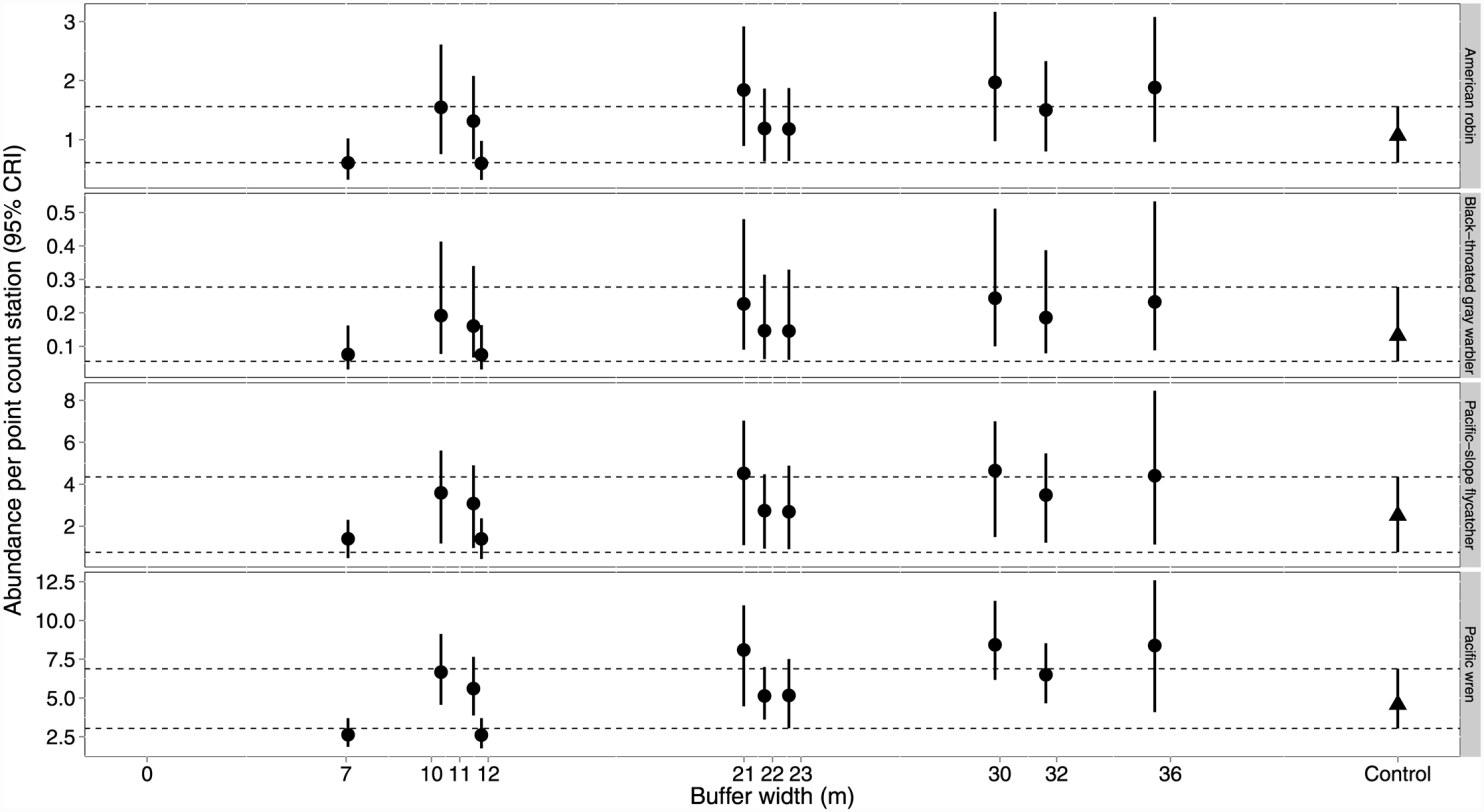
Buffer width influence on riparian associates. Site level abundance (95% confidence interval) of the four riparian associates plotted against site specific buffer width. Estimates were calculated from a model with a random site-level effect but no covariates. Control site species richness and abundance are provided on the right side (triangle) of each graphic. Intervals are confidence intervals, and not credibility intervals. Estimates for all sites were averaged across 1995–2004. Horizontal lines extending from the upper and lower bounds of the confidence intervals for the control sites are provided as reference lines.

Using the *Random Effects model*, we compared species richness and total avian abundance across buffer widths (Fig 7). Averaged across all years post-treatment, richness was generally similar between various width buffers and Controls except for lower richness on a very narrow buffer and greater richness on a wider buffer (Fig 7). Abundance was less than controls on two relatively narrow buffers and greater than controls on one wider buffer (Fig 7). For all species associated with riparian habitats (Pacific-slope flycatcher, Pacific wren, black-throated gray warbler, and American robin; Fig 8), overlap occurred between the credible intervals between controls and all stands regardless of width.

## Discussion

Buffers are often employed to conserve ecological functions in riparian ecosystems. However, considerable uncertainty exists with regards to species and community responses to riparian buffers over long time frames. For example, all studies included in Marczak et al.’s [28] meta-analysis were short-term (<5 years following forest harvest). Consequently, Marczak et al. recommended that results be viewed with “caution” (but see [33]). Over periods >5 years, species may be lost or may colonize riparian buffers, a pattern that may not be evident in short-term studies [28]. For example, migratory philopatric and territorial forest-associated species returning to their previous years’ territory may pack into the remaining habitat in the forested buffer, resulting in an increase in abundance immediately post-harvest but with a gradual reduction in density as birds sort out territorial boundaries. We found no short-term increase in avian abundance following our treatments and therefore no support for the packing hypothesis. Delayed colonization or extinction within a buffer due to gradual changes in the buffer plant community is an alternative prediction. For example, edge effects created by clearcutting the forest adjacent to riparian buffers can penetrate as much as 40 m into buffers [66], resulting in greater risk of blow-down, larger quantities of downed wood, and other structural and compositional forest changes [67, 68]. Edge effects can continue to influence forest structure and composition for upwards of 15 years post-harvest [67]. In our study, bird species richness and probability of individual species occupancy continued to increase between immediate post-harvest surveys and 10 year post-harvest surveys with no similar evidence for local species extinction over the same time period. In addition, this pattern appeared to be driven primarily by the treatments and not by other structure or compositional changes within the buffer. Because increase in species richness on buffer treatments was gradual‒may well continue beyond the time frame of this experiment‒the treatment effect (buffer width) on species turnover did not become pronounced until 10 years post-harvest. This result suggests that short-term results may not be reflective of long-term population and community responses to riparian buffers.

Using an experimental approach, we found no evidence for a short- or long-term change in estimated total avian abundance among riparian buffer treatments, regardless of the year compared. Similarly, we did not find any site-level loss of species (local-extinction) due to buffer treatments. Instead, turnover in the avian community on both the Narrow and Wide treatments resulted in the addition of species. As a result of this increase in richness on the two buffer treatments, treatments were more similar to each other in species composition than either was to the control. Many species had twice the odds of occupying treatment sites compared to the control. For most species, strong evidence for an increase in probability of occupancy on treatments relative to the controls did not become evident until ~10 years post-harvest, suggesting that colonization was occurring over an extended period of time. The change in the avian community within the riparian buffers on the treatments post-harvest was driven by the colonization of early successional species such as spotted towhee (*Pipilo maculatus*) and song sparrow (*Melospiza melodia*) and edge species like the northern flicker (*Colaptes auratus*) and olive-sided flycatcher (*Contopus cooperi*). The harvest resulted in more varied forest conditions relative to controls–the buffers contained forest, edge and early successional conditions–which, in turn, resulted in an increase in the detections of edge and open habitat species. Potential competitive interactions among the new species assemblages within riparian buffers were not measured, and represent an important topic for future research.

Relatively few studies differentiate effects of buffer width from vegetation composition and structure. Although clear differences in width existed in our buffer treatments, we also had considerable variability within and among our treatments. This variability allowed us to assess associations between buffer width and tree and shrub characteristics and avian species abundance and occupancy (this analysis did not include controls). On treatment units, we found weak evidence for a positive relationship between total avian abundance and buffer width. At the same time, we found little evidence for effects of shrub and tree covariates on abundance. This result suggests that buffer width alone is responsible for nearly all of the positive patterns we observed. Perry et al. [37] examined the effects of both forest structure and buffer width on species occupancy in the southeastern U.S. and found that, for many species, both covariates were important. However, Perry et al. examined the structure of the surrounding forests (not that of the riparian buffer) on the avian community in the buffer. In our study, forest adjacent to the riparian buffer was clearcut on all treatments and as a consequence, we examined forest composition/structure covariates within the riparian buffer and not in the adjacent harvest unit.

Estimates from a model with a random site-level effect, but no covariates, did not indicate a species richness or total abundance threshold in buffer width. These results do not provide evidence for increased species richness and abundance in forested buffers ≥ 21m when compared to controls. However, some evidence existed for reduced abundance and richness on a few sites with buffers ≤ 12m. Some sites with very narrow buffers (<12m) appear to have similar total avian abundance and richness to controls, a result which suggests considerable variation in avian response even at the narrowest buffer widths. Because we were unable to identify other vegetation covariates to explain variation in this response, we recommend research focused on identifying those mechanisms responsible for variation in narrow buffer effects. This information can direct site-specific prescriptions (e.g., provide quantitative targets) for maintaining avian abundance and richness when narrow buffers are desired [27].

When establishing buffer guidelines, agencies rarely differentiate between retaining organisms at their original abundance and simply maintaining species occupancy [28, 69]. In addition, few studies have identified which species are more abundant in riparian zones when compared to adjacent uplands. In our previous research [38], we identified “riparian associates” by comparing the relative abundance of all species in un-harvested riparian to upland habitats. This comparison identified four species that were more abundant in riparian habitats, the Pacific wren, Pacific-slope flycatcher, black-throated gray warbler and American robin [38]. This result is supported, in part, by other studies (e.g., [70]). The black-throated gray warbler, for example, forages and nests almost exclusively in deciduous trees or mixtures of deciduous and conifer trees [71, 72] which are most abundant in the riparian zone in this region [73]. Also, when compared to adjacent upslope conifer dominated habitats, Pacific-slope flycatchers in riparian habitats are more likely to attract mates, pair earlier, and have higher fecundity [74]. Despite the disproportionate use of riparian environments, we found no evidence that Narrow or Wide buffer treatments reduced abundance of these species relative to the controls. When attempting to identify buffer width thresholds for riparian associates, only the Pacific wren demonstrated very weak evidence for reduced abundance on two of the Narrow sites. Our results suggest that the riparian buffer guidelines in the Pacific region are close to the minimum needed to maintain the abundance of birds associated with forested riparian habitat. At the landscape scale, these buffers are likely more than adequate for maintaining these species. For example, on a landscape managed primarily for wood production, many areas will have young to mature stands (depending on harvest rotation) adjacent to riparian areas in addition to forested riparian buffers adjacent to recently harvested stands.

Additional factors may mediate effectiveness of riparian buffers. For example, landscape context beyond the riparian buffer can influence abundance of species within the buffer [68, 75, 76]. Characteristics of the landscape matrix, particularly amount of urban development surrounding a forest, can be better predictors of avian community composition than forest buffer width [77, 78]. Our study sites were embedded in large contiguous blocks of commercial or state forest properties (primarily in blocks > 30,000 ha) with little urban development. Other studies have classified landscapes similar to ours as “wildlands” [79] where the human footprint is relatively low [24]. In this context, landscape structure (composition and configuration) typically explains a relatively small amount of the variation in avian species abundance and species’ abundances are generally greater in more heterogeneous landscapes [80]. We note that including a random site-level effect in our model incorporates heterogeneity resulting from unmodeled site-level variation, including differences in landscape context that might be present.

Also, we did not evaluate effects of riparian buffers on avian reproduction and survival. We acknowledge the possibility that birds within narrow riparian buffers or forest fragments may not reproduce as successfully as those located in large blocks of intact forests [81, 82]. However, a relationship between reduced fecundity and habitat fragmented may not hold in all western riparian forests [83, 84]. For example, geographical differences may be associated with the occurrence of brown-headed cowbirds (*Molothrus ater*), a common brood parasite in eastern U.S. forests but one rarely encountered in some western forests [85, 86]. Assessing fitness consequences of different buffer configurations remains a critical information need given widespread implementation of buffers as beneficial conservation practices [87, 88].

Are current riparian buffer guidelines adequate for maintaining riparian-associated species? In a quantitative review of riparian buffer width guidelines and regulations from Canada and the United States, average buffer width varied from 15.1–29.0 m [26]. This variation was due to type and size of water body (lake, stream, wetland, etc.) being buffered. The average width varied geographically, with larger buffers in Canada and particularly narrow buffers in the Southeastern United States [26]. In addition, buffer width guidelines will vary depending on the biotic and abiotic objectives of the guideline or political considerations. Although forested buffers can be established to maintain species associated with aquatic and riparian conditions [89], other factors such as minimizing sedimentation [90], moderating stream temperature and light penetration [91], and maintaining riparian vegetation [67] and input of large organic debris [92] are often considered. In the Pacific region, average buffer width on small and large permanent streams ranged from 22.7–24.3 m [26]. These guidelines for this region are within the range of buffers included in our study. They are also within a range where we observed no evidence for avian species loss or for a decline in species abundance (including abundance of riparian associated species). We note that, depending on the landscape context, land owner, and individual forester, considerable operational variability will occur when riparian buffers are established. In contrast to our results, several authors have suggested that buffers ≥ 100 m are needed to maintain the complete pre-harvest avian community [93–95]; others have suggested that buffers ≥ 60 m or even narrower are needed to maintain the pre-harvest avian community [31, 32]. The relationship between buffer width and avian abundance or species composition appears to vary geographically, and it appears that wider buffers are needed in eastern deciduous forests than in the relatively wet coastal coniferous forests. Why these regional differences occur is unclear to us and merits future study.

## Supporting Information

S1 Fig Study site locations. Distribution of experimental riparian buffer study sites by treatment in western Washington, USA, 1993–2004.
(EPS)

S2 Fig Species level vegetation and buffer effects. Effect (95% credible interval) of vegetation (shrub and tree abundance) and buffer width covariates on the probability of species occupancy (circles) and abundance (triangles). This analysis disregards treatment assignments and takes advantage of the variation in the covariates within and among the two buffer treatments to examine their relative effect on site level occupancy. A solid symbol indicates 95% CRI do not overlap 0; an open symbol indicates that the 95% CRI does include 0. Bird species acronyms are provided in S1 Table.
(EPS)

S1 Table Species detections. Detections by species, year, and riparian buffer treatment, western Washington, USA, 1993, 1995–1996, and 2003–2004. C = Control, N = Narrow, and W = Wide prescriptions, respectively.
(DOCX)

S2 Table Treatment effects on detection. Median effect (95% credibility interval) of three riparian buffer treatments on detection probabilities for 28 species, western Washington, USA, 1993, 1995–1996, and 2003–2004. We averaged treatment effects across all 5 years.
(DOCX)

S3 Table Treatment effects on capture probabilities. Median effect (95% credibility interval) of three riparian buffer treatments on capture probabilities for 28 species, western Washington, USA, 1993, 1995–1996, and 2003–2004. We averaged treatment effects across all 5 years.
(DOCX)

S1 Text Code. R code and data for the MCMC implementation of the hierarchical community model and average predictive comparisons of avian species richness, western Washington, USA, 1993–2004.
(DOCX)

S2 Text Goodness of fit. Posterior predictive checks (Bayesian p-values) to assess goodness of fit for Bayesian models of avian responses, western Washington, USA, 1993–2004.
(DOCX)
