# Supplementary material for: Breeding Bird Community Continues to Colonize Riparian Buffers Ten Years after Harvest
Source: PLoS One. 2015 Dec 4;10(12):e0143241. doi: 10.1371/journal.pone.0143241 (PMC4670142; doi:10.1371/journal.pone.0143241)
Supplement: S1 Table — Detections by species, year, and riparian buffer treatment, western Washington, USA, 1993, 1995–1996, and 2003–2004. C = Control, N = Narrow, and W = Wide prescriptions, respectively. (DOCX) [file pone.0143241.s003.docx]

**S1 Table.** Number of detections by species, year, and riparian buffer treatment, western Washington, USA, 1993, 1995-1996, and 2003-2004. C = Control, N = Narrow, and W = Wide prescriptions, respectively.

|  |  | 1993 | | | 1995/1996 | | | 2003/2004 | | |  |
| --- | --- | --- | --- | --- | --- | --- | --- | --- | --- | --- | --- |
| Species | Code | C | N | W | C | N | W | C | N | W | TOTAL |
| Rufous hummingbird  *Selasphorus rufus* | RUHU | 1 | 1 | 3 | 5 | 10 | 12 | 5 | 24 | 18 | 79 |
| Red-breasted sapsucker  *Sphyrapicus ruber* | RBSA | 0 | 0 | 1 | 0 | 3 | 6 | 0 | 13 | 16 | 39 |
| Hairy woodpecker  *Picoides villosus* | HAWO | 0 | 2 | 1 | 13 | 15 | 8 | 6 | 11 | 8 | 64 |
| Northern flicker  *Colaptes auratus* | NOFL | 0 | 0 | 0 | 0 | 1 | 0 | 0 | 6 | 6 | 13 |
| Olive-sided flycatcher  *Contopus cooperi* | OSFL | 0 | 0 | 0 | 0 | 7 | 4 | 0 | 6 | 3 | 20 |
| Hammond's flycatcher  *Empidonax hammondii* | HAFL | 0 | 1 | 1 | 3 | 5 | 7 | 2 | 0 | 1 | 20 |
| Pacfic-slope flycatcher  *Empidonax difficilis* | PSFL | 21 | 21 | 11 | 44 | 50 | 34 | 49 | 33 | 32 | 295 |
| Hutton's vireo  *Vireo huttoni* | HUVI | 0 | 0 | 0 | 2 | 4 | 1 | 1 | 3 | 0 | 11 |
| Warbling vireo  *Vireo gilvus* | WAVI | 2 | 0 | 0 | 5 | 20 | 16 | 2 | 17 | 3 | 65 |
| Steller's jay  *Cyanocitta stelleri* | STJA | 3 | 1 | 0 | 6 | 7 | 6 | 5 | 18 | 23 | 69 |
| Chestnut-backed chickadee  *Poecile rufescens* | CBCH | 21 | 25 | 17 | 25 | 18 | 20 | 32 | 31 | 28 | 217 |
| Brown creeper  *Certhia americana* | BRCR | 3 | 3 | 2 | 6 | 1 | 5 | 20 | 0 | 1 | 41 |
| Winter wren  *Troglodytes troglodytes* | WIWR | 21 | 26 | 18 | 44 | 52 | 38 | 52 | 41 | 34 | 326 |
| Golden-crowned kinglet  *Regulus satrapa* | GCKI | 9 | 11 | 7 | 15 | 2 | 2 | 29 | 4 | 8 | 87 |
| Varied thrush  *Ixoreus naevius* | VATH | 0 | 2 | 0 | 3 | 0 | 1 | 11 | 0 | 2 | 19 |
| Swainson's thrush  *Catharus ustulatus* | SWTH | 4 | 4 | 3 | 14 | 8 | 13 | 20 | 18 | 21 | 105 |
| American robin  *Turdus migratorius* | AMRO | 7 | 0 | 1 | 12 | 22 | 13 | 11 | 16 | 14 | 96 |
| Cedar waxwing  Bombycilla cedrorum | CEWA | 0 | 0 | 0 | 0 | 7 | 4 | 1 | 17 | 6 | 35 |
| Hermit/Townsend's warbler  *Dendroica occidentalis/townsendi* | HETO | 3 | 4 | 1 | 3 | 0 | 1 | 0 | 0 | 0 | 12 |
| Black-throated gray warbler  *Dendroica nigrescens* | BTYW | 3 | 2 | 1 | 4 | 0 | 0 | 2 | 4 | 1 | 17 |
| Macgillivray's warbler  *Oporornis tolmei* | MGWA | 0 | 0 | 0 | 0 | 0 | 0 | 1 | 11 | 4 | 16 |
| Wilson's warbler  *Wilsonia pusilla* | WIWA | 10 | 4 | 7 | 26 | 15 | 28 | 22 | 38 | 29 | 179 |
| Western tanager  *Piranga ludoviciana* | WETA | 1 | 0 | 2 | 2 | 4 | 12 | 6 | 13 | 11 | 51 |
| Spotted towhee  *Pipilo maculatus* | SPTO | 0 | 0 | 0 | 0 | 0 | 0 | 0 | 15 | 3 | 18 |
| Song sparrow  *Melospiza melodia* | SOSP | 0 | 0 | 0 | 0 | 26 | 11 | 0 | 31 | 5 | 73 |
| Dark-eyed junco  *Junco hyemalis* | DEJU | 0 | 0 | 0 | 5 | 27 | 11 | 0 | 1 | 1 | 45 |
| Black-headed grosbeak  *Pheucticus melanocephalus* | BHGR | 0 | 0 | 0 | 3 | 9 | 2 | 2 | 12 | 11 | 39 |
| Evening grosbeak  *Coccothraustes vespertinus* | EVGR | 1 | 1 | 0 | 0 | 1 | 4 | 3 | 2 | 1 | 13 |
| TOTAL |  | 110 | 108 | 76 | 240 | 314 | 259 | 282 | 385 | 290 | 2064 |
