# Supplementary material for: Breeding Bird Community Continues to Colonize Riparian Buffers Ten Years after Harvest
Source: PLoS One. 2015 Dec 4;10(12):e0143241. doi: 10.1371/journal.pone.0143241 (PMC4670142; doi:10.1371/journal.pone.0143241)
Supplement: S2 Table — Median effect (95% credibility interval) of three riparian buffer treatments on detection probabilities for 28 species, western Washington, USA, 1993, 1995–1996, and 2003–2004. We averaged treatment effects across all 5 years. (DOCX) [file pone.0143241.s004.docx]

**S2 Table.** Median effect (95% credibility interval) of three riparian buffer treatments on detection probabilities for 28 species, western Washington, USA, 1993, 1995-1996, and 2003-2004. Treatment effects were averaged across all 5 years.

|  | Control | | Modified | | State | |
| --- | --- | --- | --- | --- | --- | --- |
| Species | Median | 95% CRI | Median | 95% CRI | Median | 95% CRI |
| RUHU | 0.16 | 0.06, 0.40 | 0.29 | 0.13, 0.54 | 0.28 | 0.13, 0.54 |
| RBSA | 0.23 | 0.05, 0.56 | 0.33 | 0.12, 0.58 | 0.27 | 0.08, 0.53 |
| HAWO | 0.20 | 0.08, 0.45 | 0.19 | 0.07, 0.43 | 0.22 | 0.09, 0.48 |
| NOFL | 0.12 | 0.01, 0.51 | 0.15 | 0.02, 0.49 | 0.11 | 0.02, 0.41 |
| OSFL | 0.31 | 0.06, 0.79 | 0.45 | 0.13, 0.85 | 0.22 | 0.06, 0.58 |
| HAFL | 0.08 | 0.02, 0.60 | 0.08 | 0.02, 0.57 | 0.04 | 0.01, 0.40 |
| PSFL | 0.85 | 0.69, 0.99 | 0.80 | 0.60, 0.99 | 0.69 | 0.47, 0.98 |
| HUVI | 0.03 | 0.00, 0.19 | 0.02 | 0, 0.13 | 0.04 | 0.01, 0.24 |
| WAVI | 0.33 | 0.12, 0.66 | 0.32 | 0.13, 0.62 | 0.57 | 0.31, 0.82 |
| STJA | 0.13 | 0.03, 0.32 | 0.33 | 0.11, 0.57 | 0.21 | 0.06, 0.42 |
| CBCH | 0.61 | 0.40, 0.80 | 0.57 | 0.35, 0.78 | 0.46 | 0.24, 0.72 |
| BRCR | 0.16 | 0.04, 0.36 | 0.07 | 0.01, 0.19 | 0.01 | 0, 0.06 |
| WIWR | 0.82 | 0.68, 0.96 | 0.76 | 0.57, 0.94 | 0.65 | 0.45, 0.90 |
| GCKI | 0.40 | 0.16, 0.63 | 0.13 | 0.04, 0.29 | 0.05 | 0.01, 0.14 |
| VATH | 0.10 | 0.03, 0.27 | 0.05 | 0.01, 0.15 | 0.01 | 0, 0.05 |
| SWTH | 0.40 | 0.11, 0.67 | 0.48 | 0.14, 0.74 | 0.22 | 0.05, 0.46 |
| AMRO | 0.24 | 0.13, 0.40 | 0.30 | 0.16, 0.49 | 0.27 | 0.15, 0.44 |
| CEWA | 0.09 | 0.01, 0.36 | 0.12 | 0.03, 0.35 | 0.21 | 0.05, 0.47 |
| HETO | 0.05 | 0.01, 0.15 | 0.03 | 0, 0.10 | 0.01 | 0, 0.04 |
| BTYW | 0.07 | 0.02, 0.20 | 0.03 | 0.01, 0.11 | 0.04 | 0.01, 0.13 |
| MGWA | 0.08 | 0.01, 0.40 | 0.08 | 0.01, 0.35 | 0.14 | 0.02, 0.45 |
| WIWA | 0.50 | 0.28, 0.71 | 0.65 | 0.41, 0.82 | 0.47 | 0.24, 0.69 |
| WETA | 0.19 | 0.06, 0.40 | 0.34 | 0.13, 0.58 | 0.20 | 0.06, 0.41 |
| SPTO | 0.07 | 0.00, 0.37 | 0.05 | 0, 0.25 | 0.16 | 0.02, 0.44 |
| SOSP | 0.24 | 0.04, 0.65 | 0.33 | 0.14, 0.57 | 0.45 | 0.25, 0.65 |
| DEJU | 0.09 | 0.01, 0.40 | 0.12 | 0.02, 0.41 | 0.26 | 0.04, 0.65 |
| BHGR | 0.17 | 0.04, 0.48 | 0.26 | 0.08, 0.61 | 0.23 | 0.08, 0.53 |
| EVGR | 0.05 | 0.01, 0.21 | 0.06 | 0.01, 0.23 | 0.03 | 0.01, 0.13 |
