# Supplementary material for: Breeding Bird Community Continues to Colonize Riparian Buffers Ten Years after Harvest
Source: PLoS One. 2015 Dec 4;10(12):e0143241. doi: 10.1371/journal.pone.0143241 (PMC4670142; doi:10.1371/journal.pone.0143241)
Supplement: S3 Table — Median effect (95% credibility interval) of three riparian buffer treatments on capture probabilities for 28 species, western Washington, USA, 1993, 1995–1996, and 2003–2004. We averaged treatment effects across all 5 years. (DOCX) [file pone.0143241.s005.docx]

**S3 Table.** Median effect (95% credibility interval) of three riparian buffer treatments on capture probabilities for 28 species, western Washington, USA, 1993, 1995-1996, and 2003-2004. Treatment effects were averaged across all 5 years.

|  | Control | | Modified | | State | |
| --- | --- | --- | --- | --- | --- | --- |
| Species | Median | 95% CRI | Median | 95% CRI | Median | 95% CRI |
| RUHU | 0.02 | 0, 0.1 | 0.05 | 0.01, 0.17 | 0.12 | 0.03,0.29 |
| RBSA | 0.01 | 0, 0.08 | 0.09 | 0.02, 0.29 | 0.18 | 0.05, 0.46 |
| HAWO | 0.02 | 0, 0.09 | 0.03 | 0, 0.12 | 0.04 | 0.01, 0.16 |
| NOFL | 0.01 | 0, 0.08 | 0.04 | 0, 0.24 | 0.06 | 0.01, 0.29 |
| OSFL | 0.02 | 0, 0.14 | 0.13 | 0.01, 0.6 | 0.13 | 0.02, 0.46 |
| HAFL | 0.03 | 0,0.21 | 0.05 | 0.01, 0.32 | 0.06 | 0.01, 0.36 |
| PSFL | 0.07 | 0.02, 0.25 | 0.04 | 0.01, 0.18 | 0.07 | 0.01, 0.27 |
| HUVI | 0.01 | 0, 0.07 | 0.01 | 0, 0.08 | 0.03 | 0, 0.18 |
| WAVI | 0.04 | 0.01, 0.2 | 0.1 | 0.02, 0.36 | 0.31 | 0.09, 0.66 |
| STJA | 0.02 | 0, 0.08 | 0.07 | 0.01, 0.26 | 0.05 | 0.01, 0.22 |
| CBCH | 0.04 | 0.01, 0.15 | 0.03 | 0, 0.13 | 0.03 | 0, 0.16 |
| BRCR | 0.02 | 0, 0.09 | 0 | 0, 0.04 | 0 | 0, 0.01 |
| WIWR | 0.05 | 0.01, 0.16 | 0.03 | 0, 0.1 | 0.05 | 0.01, 0.18 |
| GCKI | 0.06 | 0.01, 0.36 | 0.01 | 0, 0.11 | 0.01 | 0, 0.11 |
| VATH | 0.03 | 0, 0.13 | 0.01 | 0, 0.09 | 0 | 0, 0.03 |
| SWTH | 0.08 | 0.02, 0.31 | 0.13 | 0.02, 0.47 | 0.05 | 0.01, 0.25 |
| AMRO | 0.02 | 0, 0.07 | 0.03 | 0, 0.13 | 0.04 | 0.01, 0.14 |
| CEWA | 0.01 | 0, 0.04 | 0.03 | 0.01, 0.14 | 0.07 | 0.02, 0.29 |
| HETO | 0.02 | 0, 0.12 | 0.01 | 0, 0.09 | 0 | 0, 0.04 |
| BTYW | 0.02 | 0, 0.13 | 0.01 | 0, 0.08 | 0.02 | 0, 0.16 |
| MGWA | 0.01 | 0, 0.07 | 0.02 | 0, 0.14 | 0.05 | 0.01, 0.23 |
| WIWA | 0.03 | 0.01, 0.12 | 0.04 | 0.01, 0.17 | 0.05 | 0.01, 0.19 |
| WETA | 0.02 | 0, 0.1 | 0.07 | 0.01, 0.28 | 0.06 | 0.01, 0.24 |
| SPTO | 0.01 | 0, 0.06 | 0.01 | 0, 0.09 | 0.06 | 0.01, 0.25 |
| SOSP | 0.01 | 0, 0.04 | 0.07 | 0.01, 0.28 | 0.12 | 0.03, 0.31 |
| DEJU | 0.02 | 0, 0.08 | 0.03 | 0, 0.13 | 0.12 | 0.02, 0.37 |
| BHGR | 0.02 | 0, 0.11 | 0.06 | 0.01, 0.35 | 0.08 | 0.02, 0.32 |
| EVGR | 0.02 | 0, 0.11 | 0.03 | 0, 0.23 | 0.02 | 0, 0.13 |
