# Supplementary material for: Breeding Bird Community Continues to Colonize Riparian Buffers Ten Years after Harvest
Source: PLoS One. 2015 Dec 4;10(12):e0143241. doi: 10.1371/journal.pone.0143241 (PMC4670142; doi:10.1371/journal.pone.0143241)
Supplement: S1 Text — R code and data for the MCMC implementation of the hierarchical community model and average predictive comparisons of avian species richness, western Washington, USA, 1993–2004. (DOCX) [file pone.0143241.s006.docx]

**S1 Text:** R code for the MCMC implementation of the hierarchical community model and average predictive comparisons of species richness, western Washington, USA, 1993-2004.

# --------------------------------------------------------------------

# Occupancy model with treatment effects

# --------------------------------------------------------------------

bird.occ.model.trt <- function(){

# Prior distributions for community-level parameters

mu.a.0 ~dnorm(0, 0.333)

mu.a.1995 ~dnorm(0, 0.5)

mu.a.1996 ~dnorm(0, 0.5)

mu.a.2003 ~dnorm(0, 0.5)

mu.a.2004 ~dnorm(0, 0.5)

mu.a.State ~dnorm(0, 0.5)

mu.a.Modified ~dnorm(0, 0.5)

mu.a.1995.State ~dnorm(0, 0.5)

mu.a.1996.State ~dnorm(0, 0.5)

mu.a.2003.State ~dnorm(0, 0.5)

mu.a.2004.State ~dnorm(0, 0.5)

mu.a.1995.Modified ~dnorm(0, 0.5)

mu.a.1996.Modified ~dnorm(0, 0.5)

mu.a.2003.Modified ~dnorm(0, 0.5)

mu.a.2004.Modified ~dnorm(0, 0.5)

mu.b.0 ~dnorm(0, 0.333)

mu.b.1995 ~dnorm(0, 0.5)

mu.b.1996 ~dnorm(0, 0.5)

mu.b.2003 ~dnorm(0, 0.5)

mu.b.2004 ~dnorm(0, 0.5)

mu.b.State ~dnorm(0, 0.5)

mu.b.Modified ~dnorm(0, 0.5)

mu.b.date ~dnorm(0, 0.5)

mu.b.date.2 ~dnorm(0, 0.5)

tau.a.0 ~dgamma(1.5,.5)

tau.a.site ~dgamma(1.5,.5)

tau.a.1995 ~dgamma(1.5,.5)

tau.a.1996 ~dgamma(1.5,.5)

tau.a.2003 ~dgamma(1.5,.5)

tau.a.2004 ~dgamma(1.5,.5)

tau.a.State ~dgamma(1.5,.5)

tau.a.Modified ~dgamma(1.5,.5)

tau.a.1995.State ~dgamma(1.5,.5)

tau.a.1996.State ~dgamma(1.5,.5)

tau.a.2003.State ~dgamma(1.5,.5)

tau.a.2004.State ~dgamma(1.5,.5)

tau.a.1995.Modified ~dgamma(1.5,.5)

tau.a.1996.Modified ~dgamma(1.5,.5)

tau.a.2003.Modified ~dgamma(1.5,.5)

tau.a.2004.Modified ~dgamma(1.5,.5)

tau.b.0 ~dgamma(1.5,.5)

tau.b.1995 ~dgamma(1.5,.5)

tau.b.1996 ~dgamma(1.5,.5)

tau.b.2003 ~dgamma(1.5,.5)

tau.b.2004 ~dgamma(1.5,.5)

tau.b.State ~dgamma(1.5,.5)

tau.b.Modified ~dgamma(1.5,.5)

tau.b.date ~dgamma(1.5,.5)

tau.b.date.2 ~dgamma(1.5,.5)

sig.a.0 <- pow(tau.a.0,-.5)

sig.a.site <- pow(tau.a.site,-.5)

sig.a.1995 <- pow(tau.a.1995,-.5)

sig.a.1996 <- pow(tau.a.1996,-.5)

sig.a.2003 <- pow(tau.a.2003,-.5)

sig.a.2004 <- pow(tau.a.2004,-.5)

sig.a.State <- pow(tau.a.State,-.5)

sig.a.Modified <- pow(tau.a.Modified,-.5)

sig.a.1995.State <- pow(tau.a.1995.State,-.5)

sig.a.1996.State <- pow(tau.a.1996.State,-.5)

sig.a.2003.State <- pow(tau.a.2003.State,-.5)

sig.a.2004.State <- pow(tau.a.2004.State,-.5)

sig.a.1995.Modified <- pow(tau.a.1995.Modified,-.5)

sig.a.1996.Modified <- pow(tau.a.1996.Modified,-.5)

sig.a.2003.Modified <- pow(tau.a.2003.Modified,-.5)

sig.a.2004.Modified <- pow(tau.a.2004.Modified,-.5)

sig.b.0 <- pow(tau.b.0,-.5)

sig.b.1995 <- pow(tau.b.1995,-.5)

sig.b.1996 <- pow(tau.b.1996,-.5)

sig.b.2003 <- pow(tau.b.2003,-.5)

sig.b.2004 <- pow(tau.b.2004,-.5)

sig.b.State <- pow(tau.b.State,-.5)

sig.b.Modified <- pow(tau.b.Modified,-.5)

sig.b.date <- pow(tau.b.date,-.5)

sig.b.date.2 <- pow(tau.b.date.2,-.5)

for (k in 1:nsite){

a.site[k] ~ dnorm(0, tau.a.site)

}

# Create priors for from the community level prior distributions

for (i in 1:nspecies) {

a.0[i] ~ dnorm(mu.a.0, tau.a.0)

a.1995[i] ~ dnorm(mu.a.1995, tau.a.1995)

a.1996[i] ~ dnorm(mu.a.1996, tau.a.1996)

a.2003[i] ~ dnorm(mu.a.2003, tau.a.2003)

a.2004[i] ~ dnorm(mu.a.2004, tau.a.2004)

a.State[i] ~ dnorm(mu.a.State, tau.a.State)

a.Modified[i] ~ dnorm(mu.a.Modified, tau.a.Modified)

a.1995.State[i] ~ dnorm(mu.a.1995.State, tau.a.1995.State)

a.1996.State[i] ~ dnorm(mu.a.1996.State, tau.a.1996.State)

a.2003.State[i] ~ dnorm(mu.a.2003.State, tau.a.2003.State)

a.2004.State[i] ~ dnorm(mu.a.2004.State, tau.a.2004.State)

a.1995.Modified[i]~ dnorm(mu.a.1995.Modified, tau.a.1995.Modified)

a.1996.Modified[i]~ dnorm(mu.a.1996.Modified, tau.a.1996.Modified)

a.2003.Modified[i]~ dnorm(mu.a.2003.Modified, tau.a.2003.Modified)

a.2004.Modified[i ~ dnorm(mu.a.2003.Modified, tau.a.2003.Modified)

b.0[i] ~ dnorm(mu.b.0, tau.b.0)

b.1995[i] ~ dnorm(mu.b.1995, tau.b.1995)

b.1996[i] ~ dnorm(mu.b.1996, tau.b.1996)

b.2003[i] ~ dnorm(mu.b.2003, tau.b.2003)

b.2004[i] ~ dnorm(mu.b.2004, tau.b.2004)

b.State[i] ~ dnorm(mu.b.State, tau.b.State)

b.Modified[i] ~ dnorm(mu.b.Modified, tau.b.Modified)

b.date[i] ~ dnorm(mu.b.date, tau.b.date)

b.date.2[i] ~ dnorm(mu.b.date.2, tau.b.date.2)

for (j in 1:nyear){

# Create a loop to estimate the true occurrence for species i.

for (k in 1:nsite) {

logit(psi[k,j,i]) <- a.0[i] + a.site[k] + a.1995[i]*year.1995[j]

+ a.1996[i]*year.1996[j] + a.2003[i]*year.2003[j] + a.2004[i]*year.2004[j]

+ a.State[i]*trt.State[k] + a.Modified[i]*trt.Modified[k]

+ a.1995.State[i]*year.1995[j]*trt.State[k]

+ a.1996.State[i]*year.1996[j]*trt.State[k]

+ a.2003.State[i]*year.2003[j]*trt.State[k]

+ a.2004.State[i]*year.2004[j]*trt.State[k]

+ a.1995.Modified[i]*year.1995[j]*trt.Modified[k]

+ a.1996.Modified[i]*year.1996[j]*trt.Modified[k]

+ a.2003.Modified[i]*year.2003[j]*trt.Modified[k]

+ a.2004.Modified[i]*year.2004[j]*trt.Modified[k]

mu.psi[k,j,i] <- min(0.999, max(psi[k,j,i], 0.001))

Z[k,j,i] ~ dbern(mu.psi[k,j,i])

# Create a loop to estimate detection

for (l in 1:nvisit[j,k]) {

logit(p[l,k,j,i]) <- b.0[i] + b.1995[i]*year.1995[j] + b.1996[i]*year.1996[j]

+ b.2003[i]*year.2003[j] + b.2004[i]*year.2004[j]

+ b.State[i]*trt.State.det[k,j] + b.Modified[i]*trt.Modified.det[k,j]

+ b.date[i]*date.matrix[j,k,l] + b.date.2[i]*date.matrix.2[j,k,l]

mu.p[l,k,j,i] <- min(0.999, max(p[l,k,j,i], 0.001))*Z[k,j,i]

X[l,k,j,i] ~ dbern(mu.p[l,k,j,i])

}

}

}

}

}

# --------------------------------------------------------------------

# Occupancy model with continuous covariates

# --------------------------------------------------------------------

bird.occ.model.cont <- function(){

# Prior distributions for community-level parameters

mu.a.0 ~dnorm(0, 0.333)

mu.a.1996 ~dnorm(0, 0.5)

mu.a.2003 ~dnorm(0, 0.5)

mu.a.2004 ~dnorm(0, 0.5)

mu.a.buffer ~dnorm(0, 0.5)

mu.a.shrub ~dnorm(0, 0.5)

mu.a.dougfir ~dnorm(0, 0.5)

mu.a.decid ~dnorm(0, 0.5)

mu.a.hemcedar ~dnorm(0, 0.5)

mu.b.0 ~dnorm(0, 0.333)

mu.b.1996 ~dnorm(0, 0.5)

mu.b.2003 ~dnorm(0, 0.5)

mu.b.2004 ~dnorm(0, 0.5)

mu.b.buffer ~dnorm(0, 0.5)

mu.b.shrub ~dnorm(0, 0.5)

mu.b.dougfir ~dnorm(0, 0.5)

mu.b.decid ~dnorm(0, 0.5)

mu.b.hemcedar ~dnorm(0, 0.5)

mu.b.date ~dnorm(0, 0.5)

mu.b.date.2 ~dnorm(0, 0.5)

tau.a.0 ~dgamma(1.5,.5)

tau.a.site ~dgamma(1.5,.5)

tau.a.1996 ~dgamma(1.5,.5)

tau.a.2003 ~dgamma(1.5,.5)

tau.a.2004 ~dgamma(1.5,.5)

tau.a.buffer ~dgamma(1.5,.5)

tau.a.shrub ~dgamma(1.5,.5)

tau.a.dougfir ~dgamma(1.5,.5)

tau.a.decid ~dgamma(1.5,.5)

tau.a.hemcedar ~dgamma(1.5,.5)

tau.b.0 ~dgamma(1.5,.5)

tau.b.1996 ~dgamma(1.5,.5)

tau.b.2003 ~dgamma(1.5,.5)

tau.b.2004 ~dgamma(1.5,.5)

tau.b.buffer ~dgamma(1.5,.5)

tau.b.shrub ~dgamma(1.5,.5)

tau.b.dougfir ~dgamma(1.5,.5)

tau.b.decid ~dgamma(1.5,.5)

tau.b.hemcedar ~dgamma(1.5,.5)

tau.b.date ~dgamma(1.5,.5)

tau.b.date.2 ~dgamma(1.5,.5)

sig.a.0 <-pow(tau.a.0,-.5)

sig.a.site <-pow(tau.a.site,-.5)

sig.a.1996 <-pow(tau.a.1996,-.5)

sig.a.2003 <-pow(tau.a.2003,-.5)

sig.a.2004 <-pow(tau.a.2004,-.5)

sig.a.buffer <-pow(tau.a.buffer,-.5)

sig.a.shrub <-pow(tau.a.shrub,-.5)

sig.a.dougfir <-pow(tau.a.dougfir,-.5)

sig.a.decid <-pow(tau.a.decid,-.5)

sig.a.hemcedar <-pow(tau.a.hemcedar,-.5)

sig.b.0 <-pow(tau.b.0,-.5)

sig.b.1996 <-pow(tau.b.1996,-.5)

sig.b.2003 <-pow(tau.b.2003,-.5)

sig.b.2004 <-pow(tau.b.2004,-.5)

sig.b.buffer <-pow(tau.b.buffer,-.5)

sig.b.shrub <-pow(tau.b.shrub,-.5)

sig.b.dougfir <-pow(tau.b.dougfir,-.5)

sig.b.decid <-pow(tau.b.decid,-.5)

sig.b.hemcedar <-pow(tau.b.hemcedar,-.5)

sig.b.date <-pow(tau.b.date,-.5)

sig.b.date.2 <-pow(tau.b.date.2,-.5)

for (k in 1:nsite){

a.site[k] ~ dnorm(0, tau.a.site)

}

# Create priors for species i from the community level prior distributions

for (i in 1:nspecies) {

a.0[i] ~ dnorm(mu.a.0, tau.a.0)

a.1996[i] ~ dnorm(mu.a.1996, tau.a.1996)

a.2003[i] ~ dnorm(mu.a.2003, tau.a.2003)

a.2004[i] ~ dnorm(mu.a.2004, tau.a.2004)

a.buffer[i] ~ dnorm(mu.a.buffer, tau.a.buffer)

a.shrub[i] ~ dnorm(mu.a.shrub, tau.a.shrub)

a.dougfir[i] ~ dnorm(mu.a.dougfir, tau.a.dougfir)

a.decid[i] ~ dnorm(mu.a.decid, tau.a.decid)

a.hemcedar[i] ~ dnorm(mu.a.hemcedar, tau.a.hemcedar)

b.0[i] ~ dnorm(mu.b.0, tau.b.0)

b.1996[i] ~ dnorm(mu.b.1996, tau.b.1996)

b.2003[i] ~ dnorm(mu.b.2003, tau.b.2003)

b.2004[i] ~ dnorm(mu.b.2004, tau.b.2004)

b.buffer[i] ~ dnorm(mu.b.buffer, tau.b.buffer)

b.shrub[i] ~ dnorm(mu.b.shrub, tau.b.shrub)

b.dougfir[i] ~ dnorm(mu.b.dougfir, tau.b.dougfir)

b.decid[i] ~ dnorm(mu.b.decid, tau.b.decid)

b.hemcedar[i] ~ dnorm(mu.b.hemcedar, tau.b.hemcedar)

b.date[i] ~ dnorm(mu.b.date, tau.b.date)

b.date.2[i] ~ dnorm(mu.b.date.2, tau.b.date.2)

for (j in 1:nyear){

# Create a loop to estimate the Z matrix true occupancy

for (k in 1:nsite) {

logit(psi[k,j,i]) <- a.0[i] + a.site[k] + a.1996[i]*year.1996[j]

+ a.2003[i]*year.2003[j] + a.2004[i]*year.2004[j]

+ a.buffer[i]*buffer.width.center.scaled[k] + a.shrub[i]*ShrubCover[k,j]

+ a.dougfir[i]*DougFir[k,j] + a.decid[i]*Deciduous[k,j]

+ a.hemcedar[i]*HemlockCedar[k,j]

mu.psi[k,j,i] <- min(0.99, max(psi[k,j,i], 0.01))

Z[k,j,i] ~ dbern(mu.psi[k,j,i])

# Create a loop to estimate detection probability

for (l in 1:nvisit.harvested[j,k]) {

logit(p[l,k,j,i]) <- b.0[i] + b.1996[i]*year.1996[j] + b.2003[i]*year.2003[j]

+ b.2004[i]*year.2004[j] + b.buffer[i]*buffer.width.center.scaled[k]

+ b.shrub[i]*ShrubCover[k,j] + b.dougfir[i]*DougFir[k,j]

+ b.decid[i]*Deciduous[k,j]+ b.hemcedar[i]*HemlockCedar[k,j]

+ b.date[i]*date.matrix[j,k,l] + b.date.2[i]*date.matrix.2[j,k,l]

mu.p[l,k,j,i] <- min(0.99, max(p[l,k,j,i], 0.01))*Z[k,j,i]

X[l,k,j,i] ~ dbern(mu.p[l,k,j,i])

}

}

}

}

}

# --------------------------------------------------------------------

# Abundance model with treatment effects

# --------------------------------------------------------------------

bird.abund.model <- function(){

# Prior distributions for community-level parameters

mu.a.0 ~dnorm(1.2, .2)

mu.a.1995 ~dnorm(0, 0.2)

mu.a.1996 ~dnorm(0, 0.2)

mu.a.2003 ~dnorm(0, 0.2)

mu.a.2004 ~dnorm(0, 0.2)

mu.a.State ~dnorm(0, 0.2)

mu.a.Modified ~dnorm(0, 0.2)

mu.a.1995.State ~dnorm(0, 0.2)

mu.a.1996.State ~dnorm(0, 0.2)

mu.a.2003.State ~dnorm(0, 0.2)

mu.a.2004.State ~dnorm(0, 0.2)

mu.a.1995.Modified ~dnorm(0, 0.2)

mu.a.1996.Modified ~dnorm(0, 0.2)

mu.a.2003.Modified ~dnorm(0, 0.2)

mu.a.2004.Modified ~dnorm(0, 0.2)

mu.b.0 ~dnorm(0, 0.333)

mu.b.1995 ~dnorm(0, 0.2)

mu.b.1996 ~dnorm(0, 0.2)

mu.b.2003 ~dnorm(0, 0.2)

mu.b.2004 ~dnorm(0, 0.2)

mu.b.State ~dnorm(0, 0.2)

mu.b.Modified ~dnorm(0, 0.2)

mu.b.date ~dnorm(0, 0.2)

mu.b.date.2 ~dnorm(0, 0.2)

tau.a.0 ~dgamma(1.5,.5)

tau.a.site ~dgamma(1.5,.5)

tau.a.1995 ~dgamma(1.5,.5)

tau.a.1996 ~dgamma(1.5,.5)

tau.a.2003 ~dgamma(1.5,.5)

tau.a.2004 ~dgamma(1.5,.5)

tau.a.State ~dgamma(1.5,.5)

tau.a.Modified ~dgamma(1.5,.5)

tau.a.1995.State ~dgamma(1.5,.5)

tau.a.1996.State ~dgamma(1.5,.5)

tau.a.2003.State ~dgamma(1.5,.5)

tau.a.2004.State ~dgamma(1.5,.5)

tau.a.1995.Modified ~dgamma(1.5,.5)

tau.a.1996.Modified ~dgamma(1.5,.5)

tau.a.2003.Modified ~dgamma(1.5,.5)

tau.a.2004.Modified ~dgamma(1.5,.5)

tau.b.0 ~dgamma(1.5,.5)

tau.b.1995 ~dgamma(1.5,.5)

tau.b.1996 ~dgamma(1.5,.5)

tau.b.2003 ~dgamma(1.5,.5)

tau.b.2004 ~dgamma(1.5,.5)

tau.b.State ~dgamma(1.5,.5)

tau.b.Modified ~dgamma(1.5,.5)

tau.b.date ~dgamma(1.5,.5)

tau.b.date.2 ~dgamma(1.5,.5)

sig.a.0 <- pow(tau.a.0, -.5)

sig.a.site <- pow(tau.a.site, -.5)

sig.a.1995 <- pow(tau.a.1995, -.5)

sig.a.1996 <- pow(tau.a.1996, -.5)

sig.a.2003 <- pow(tau.a.2003, -.5)

sig.a.2004 <- pow(tau.a.2004, -.5)

sig.a.State <- pow(tau.a.State, -.5)

sig.a.Modified <- pow(tau.a.Modified, -.5)

sig.a.1995.State <- pow(tau.a.1995.State, -.5)

sig.a.1996.State <- pow(tau.a.1996.State, -.5)

sig.a.2003.State <- pow(tau.a.2003.State, -.5)

sig.a.2004.State <- pow(tau.a.2004.State, -.5)

sig.a.1995.Modified <- pow(tau.a.1995.Modified, -.5)

sig.a.1996.Modified <- pow(tau.a.1996.Modified, -.5)

sig.a.2003.Modified <- pow(tau.a.2003.Modified, -.5)

sig.a.2004.Modified <- pow(tau.a.2004.Modified, -.5)

sig.b.0 <- pow(tau.b.0, -.5)

sig.b.1995 <- pow(tau.b.1995, -.5)

sig.b.1996 <- pow(tau.b.1996, -.5)

sig.b.2003 <- pow(tau.b.2003, -.5)

sig.b.2004 <- pow(tau.b.2004, -.5)

sig.b.State <- pow(tau.b.State, -.5)

sig.b.Modified <- pow(tau.b.Modified, -.5)

sig.b.date <- pow(tau.b.date, -.5)

sig.b.date.2 <- pow(tau.b.date.2, -.5)

for (k in 1:nsite){

a.site[k] ~ dnorm(0, tau.a.site)

}

# Create priors for species i from the community level prior distributions

for (i in 1:nspecies) {

a.0[i] ~ dnorm(mu.a.0, tau.a.0)

a.1995[i] ~ dnorm(mu.a.1995, tau.a.1995)

a.1996[i] ~ dnorm(mu.a.1996, tau.a.1996)

a.2003[i] ~ dnorm(mu.a.2003, tau.a.2003)

a.2004[i] ~ dnorm(mu.a.2004, tau.a.2004)

a.State[i] ~ dnorm(mu.a.State, tau.a.State)

a.Modified[i] ~ dnorm(mu.a.Modified, tau.a.Modified)

a.1995.State[i] ~ dnorm(mu.a.1995.State, tau.a.1995.State)

a.1996.State[i] ~ dnorm(mu.a.1996.State, tau.a.1996.State)

a.2003.State[i] ~ dnorm(mu.a.2003.State, tau.a.2003.State)

a.2004.State[i] ~ dnorm(mu.a.2004.State, tau.a.2004.State)

a.1995.Modified[i] ~ dnorm(mu.a.1995.Modified, tau.a.1995.Modified)

a.1996.Modified[i] ~ dnorm(mu.a.1996.Modified, tau.a.1996.Modified)

a.2003.Modified[i] ~ dnorm(mu.a.2003.Modified, tau.a.2003.Modified)

a.2004.Modified[i] ~ dnorm(mu.a.2003.Modified, tau.a.2003.Modified)

b.0[i] ~ dnorm(mu.b.0, tau.b.0)

b.1995[i] ~ dnorm(mu.b.1995, tau.b.1995)

b.1996[i] ~ dnorm(mu.b.1996, tau.b.1996)

b.2003[i] ~ dnorm(mu.b.2003, tau.b.2003)

b.2004[i] ~ dnorm(mu.b.2004, tau.b.2004)

b.State[i] ~ dnorm(mu.b.State, tau.b.State)

b.Modified[i] ~ dnorm(mu.b.Modified, tau.b.Modified)

b.date[i] ~ dnorm(mu.b.date, tau.b.date)

b.date.2[i] ~ dnorm(mu.b.date.2, tau.b.date.2)

for (j in 1:nyear){

# Create a loop to estimate abundance

for (k in 1:nsite) {

log(lambda[k,j,i]) <- a.0[i] + a.site[k] + a.1995[i]*year.1995[j]

+ a.1996[i]*year.1996[j] + a.2003[i]*year.2003[j] + a.2004[i]*year.2004[j]

+ a.State[i]*trt.State[k] + a.Modified[i]*trt.Modified[k]

+ a.1995.State[i]*year.1995[j]*trt.State[k]

+ a.1996.State[i]*year.1996[j]*trt.State[k]

+ a.2003.State[i]*year.2003[j]*trt.State[k]

+ a.2004.State[i]*year.2004[j]*trt.State[k]

+ a.1995.Modified[i]*year.1995[j]*trt.Modified[k]

+ a.1996.Modified[i]*year.1996[j]*trt.Modified[k]

+ a.2003.Modified[i]*year.2003[j]*trt.Modified[k]

+ a.2004.Modified[i]*year.2004[j]*trt.Modified[k]

N[k,j,i] ~ dpois(lambda[k,j,i])

# Create a loop to estimate detection probability.

for (l in 1:nvisit[j,k]) {

logit(p[l,k,j,i]) <- b.0[i] + b.1995[i]*year.1995[j] + b.1996[i]*year.1996[j]

+ b.2003[i]*year.2003[j] + b.2004[i]*year.2004[j]

+ b.State[i]*trt.State.det[k,j]+ b.Modified[i]*trt.Modified.det[k,j]

+ b.date[i]*date.matrix[j,k,l] + b.date.2[i]*date.matrix.2[j,k,l]

mu.p[l,k,j,i] <- min(0.999, max(p[l,k,j,i], 0.001))

y.obs[l,k,j,i] ~ dbin(mu.p[l,k,j,i], N[k,j,i])

}

}

}

}

}

# --------------------------------------------------------------------

# Abundance model with continuous covariates

# --------------------------------------------------------------------

bird.abund.model <- function(){

# Prior distributions for community-level parameters

mu.a.0 ~dnorm(1.2, .2)

mu.a.1996 ~dnorm(0, 0.2)

mu.a.2003 ~dnorm(0, 0.2)

mu.a.2004 ~dnorm(0, 0.2)

mu.a.buffer ~dnorm(0, 0.2)

mu.a.shrub ~dnorm(0, 0.2)

mu.a.dougfir ~dnorm(0, 0.2)

mu.a.decid ~dnorm(0, 0.2)

mu.a.hemcedar ~dnorm(0, 0.2)

mu.b.0 ~dnorm(0, 0.333)

mu.b.1996 ~dnorm(0, 0.2)

mu.b.2003 ~dnorm(0, 0.2)

mu.b.2004 ~dnorm(0, 0.2)

mu.b.buffer ~dnorm(0, 0.2)

mu.b.shrub ~dnorm(0, 0.2)

mu.b.dougfir ~dnorm(0, 0.2)

mu.b.decid ~dnorm(0, 0.2)

mu.b.hemcedar ~dnorm(0, 0.2)

mu.b.date ~dnorm(0, 0.2)

mu.b.date.2 ~dnorm(0, 0.2)

tau.a.0 ~dgamma(1.5,.5)

tau.a.site ~dgamma(1.5,.5)

tau.a.1996 ~dgamma(1.5,.5)

tau.a.2003 ~dgamma(1.5,.5)

tau.a.2004 ~dgamma(1.5,.5)

tau.a.buffer ~dgamma(1.5,.5)

tau.a.shrub ~dgamma(1.5,.5)

tau.a.dougfir ~dgamma(1.5,.5)

tau.a.decid ~dgamma(1.5,.5)

tau.a.hemcedar ~dgamma(1.5,.5)

tau.b.0 ~dgamma(1.5,.5)

tau.b.1996 ~dgamma(1.5,.5)

tau.b.2003 ~dgamma(1.5,.5)

tau.b.2004 ~dgamma(1.5,.5)

tau.b.buffer ~dgamma(1.5,.5)

tau.b.shrub ~dgamma(1.5,.5)

tau.b.dougfir ~dgamma(1.5,.5)

tau.b.decid ~dgamma(1.5,.5)

tau.b.hemcedar ~dgamma(1.5,.5)

tau.b.date ~dgamma(1.5,.5)

tau.b.date.2 ~dgamma(1.5,.5)

sig.a.0 <-pow(tau.a.0,-.5)

sig.a.site <-pow(tau.a.site,-.5)

sig.a.1996 <-pow(tau.a.1996,-.5)

sig.a.2003 <-pow(tau.a.2003,-.5)

sig.a.2004 <-pow(tau.a.2004,-.5)

sig.a.buffer <-pow(tau.a.buffer,-.5)

sig.a.shrub <-pow(tau.a.shrub,-.5)

sig.a.dougfir <-pow(tau.a.dougfir,-.5)

sig.a.decid <-pow(tau.a.decid,-.5)

sig.a.hemcedar <-pow(tau.a.hemcedar,-.5)

sig.b.0 <-pow(tau.b.0,-.5)

sig.b.1996 <-pow(tau.b.1996,-.5)

sig.b.2003 <-pow(tau.b.2003,-.5)

sig.b.2004 <-pow(tau.b.2004,-.5)

sig.b.buffer <-pow(tau.b.buffer, -.5)

sig.b.shrub <-pow(tau.b.shrub, -.5)

sig.b.dougfir <-pow(tau.b.dougfir, -.5)

sig.b.decid <-pow(tau.b.decid, -.5)

sig.b.hemcedar <-pow(tau.b.hemcedar,-.5)

sig.b.date <-pow(tau.b.date,-.5)

sig.b.date.2 <-pow(tau.b.date.2,-.5)

for (k in 1:nsite){

a.site[k] ~ dnorm(0, tau.a.site)

}

# Create priors for species i from the community level prior distributions

for (i in 1:nspecies) {

a.0[i] ~ dnorm(mu.a.0, tau.a.0)

a.1996[i] ~ dnorm(mu.a.1996, tau.a.1996)

a.2003[i] ~ dnorm(mu.a.2003, tau.a.2003)

a.2004[i] ~ dnorm(mu.a.2004, tau.a.2004)

a.buffer[i] ~ dnorm(mu.a.buffer, tau.a.buffer)

a.shrub[i] ~ dnorm(mu.a.shrub, tau.a.shrub)

a.dougfir[i] ~ dnorm(mu.a.dougfir, tau.a.dougfir)

a.decid[i] ~ dnorm(mu.a.decid, tau.a.decid)

a.hemcedar[i] ~ dnorm(mu.a.hemcedar, tau.a.hemcedar)

b.0[i] ~ dnorm(mu.b.0, tau.b.0)

b.1996[i] ~ dnorm(mu.b.1996, tau.b.1996)

b.2003[i] ~ dnorm(mu.b.2003, tau.b.2003)

b.2004[i] ~ dnorm(mu.b.2004, tau.b.2004)

b.buffer[i] ~ dnorm(mu.b.buffer, tau.b.buffer)

b.shrub[i] ~ dnorm(mu.b.shrub, tau.b.shrub)

b.dougfir[i] ~ dnorm(mu.b.dougfir, tau.b.dougfir)

b.decid[i] ~ dnorm(mu.b.decid, tau.b.decid)

b.hemcedar[i] ~ dnorm(mu.b.hemcedar, tau.b.hemcedar)

b.date[i] ~ dnorm(mu.b.date, tau.b.date)

b.date.2[i] ~ dnorm(mu.b.date.2, tau.b.date.2)

for (j in 1:nyear){

# Create a loop to estimate the true abundance.

for (k in 1:nsite) {

log(lambda[k,j,i]) <- a.0[i] + a.site[k] + a.1996[i]*year.1996[j]

+ a.2003[i]*year.2003[j] + a.2004[i]*year.2004[j]

+ a.buffer[i]*buffer.width.center.scaled[k]

+ a.shrub[i]*ShrubCover[k,j] + a.dougfir[i]*DougFir[k,j]

+ a.decid[i]*Deciduous[k,j] + a.hemcedar[i]*HemlockCedar[k,j]

N[k,j,i] ~ dpois(lambda[k,j,i])

# Create a loop to estimate detection probability.

for (l in 1:nvisit.harvested[j,k]) {

logit(p[l,k,j,i]) <- b.0[i] + b.1996[i]*year.1996[j] + b.2003[i]*year.2003[j]

+ b.2004[i]*year.2004[j] + b.buffer[i]*buffer.width.center.scaled[k]

+ b.shrub[i]*ShrubCover[k,j] + b.dougfir[i]*DougFir[k,j]

+ b.decid[i]*Deciduous[k,j]+ b.hemcedar[i]*HemlockCedar[k,j]

+ b.date[i]*date.matrix[j,k,l] + b.date.2[i]*date.matrix.2[j,k,l]

mu.p[l,k,j,i] <- min(0.999, max(p[l,k,j,i], 0.001))

y.obs[l,k,j,i] ~ dbin(mu.p[l,k,j,i], N[k,j,i])

}

}

}

}

}
