# Supplementary material for: Breeding Bird Community Continues to Colonize Riparian Buffers Ten Years after Harvest
Source: PLoS One. 2015 Dec 4;10(12):e0143241. doi: 10.1371/journal.pone.0143241 (PMC4670142; doi:10.1371/journal.pone.0143241)
Supplement: S2 Text — Posterior predictive checks (Bayesian p-values) to assess goodness of fit for Bayesian models of avian responses, western Washington, USA, 1993–2004. (DOCX) [file pone.0143241.s007.docx]

**S2 Text. Posterior predictive checks (Bayesian p-values) to assess goodness of fit for Bayesian models, western Washington, USA, 1993-2004.**

To assess consistency between data and our models, we used posterior predictive checks (Gelman and Hill 2007). For our posterior predictive checks, we simulated a data set for each of our MCMC posterior samples of true occupancy and observed occupancy for the occupancy models and true abundance and observed abundance for the abundance models. After simulating new data from the model, we calculated the proportion of visits with at least one detection from the occupancy model and the average number of observed individuals for the abundance model. We then calculated the proportion of times that the simulated proportion and average exceeded the observed from our dataset. Values close to 0 or 1 suggest lack of fit between the data and the model, while values close to 0.5 suggest agreement between the data and the model. Only PSFL in the abundance model with continuous covariates had a p-value that indicated any lack of fit. Otherwise, we did not find any evidence suggesting lack-of-fit between the data and our model using this statistic. We included a table of Bayesian p-values comparing the proportion of times that the observed values were larger than the simulated values (Table S2.1).

Table S2.1: Bayesian p-values to compare the the proportion of times that the observed values were larger than the simulated values.

| Species | **Occupancy models** | | **Abundance models** | |
| --- | --- | --- | --- | --- |
|  | Treatment | Continuous | Treatment | Continuous |
| AMRO | 0.47 | 0.48 | 0.26 | 0.46 |
| BGWA | 0.44 | 0.34 | 0.36 | 0.69 |
| BHGR | 0.52 | 0.43 | 0.34 | 0.46 |
| BRCR | 0.49 | 0.44 | 0.44 | 0.41 |
| CBCH | 0.57 | 0.54 | 0.17 | 0.52 |
| CEWA | 0.57 | 0.49 | 0.44 | 0.44 |
| DEJU | 0.59 | 0.53 | 0.30 | 0.21 |
| EVGR | 0.41 | 0.42 | 0.37 | 0.69 |
| GCKI | 0.47 | 0.43 | 0.20 | 0.75 |
| HAFL | 0.39 | 0.42 | 0.42 | 0.21 |
| HAWO | 0.49 | 0.50 | 0.33 | 0.28 |
| HETO | 0.44 | 0.25 | 0.40 | 0.32 |
| HUVI | 0.42 | 0.44 | 0.37 | 0.40 |
| MGWA | 0.52 | 0.46 | 0.49 | 0.47 |
| NOFL | 0.51 | 0.39 | 0.47 | 0.46 |
| OSFL | 0.45 | 0.27 | 0.45 | 0.27 |
| PSFL | 0.56 | 0.63 | 0.21 | 0.03 |
| RBSA | 0.61 | 0.43 | 0.46 | 0.37 |
| RUHU | 0.45 | 0.49 | 0.33 | 0.28 |
| SOSP | 0.74 | 0.54 | 0.69 | 0.30 |
| SPTO | 0.56 | 0.41 | 0.59 | 0.50 |
| STJA | 0.48 | 0.43 | 0.43 | 0.53 |
| SWTH | 0.52 | 0.57 | 0.37 | 0.51 |
| VATH | 0.45 | 0.38 | 0.51 | 0.53 |
| WAVI | 0.52 | 0.50 | 0.27 | 0.12 |
| WETA | 0.56 | 0.50 | 0.45 | 0.38 |
| WIWA | 0.53 | 0.63 | 0.31 | 0.82 |
| WIWR | 0.58 | 0.63 | 0.15 | 0.10 |
